# A Two-Step Mechanism for Cell Fate Decision by Coordination of Nuclear and Mitochondrial p53 Activities

**DOI:** 10.1371/journal.pone.0038164

**Published:** 2012-06-05

**Authors:** Xiao-Jun Tian, Feng Liu, Xiao-Peng Zhang, Jun Li, Wei Wang

**Affiliations:** National Laboratory of Solid State Microstructures and Department of Physics, Nanjing University, Nanjing, China; Institute of Pathology, Germany

## Abstract

The tumor suppressor p53 has a crucial role in the DNA damage response. Here, we proposed an integrated model of the p53 network and explored how the nuclear and mitochondrial p53 pathways are coordinated to determine cell fates after 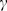-irradiation in radiosensitive tissues. Using numerical simulations, we found that depending on the extent of DNA damage, cells may survive, commit apoptosis after cell cycle arrest, or undergo apoptosis soon after irradiation. There exists a large cell-to-cell variability in outcome because of stochasticity in the generation and repair of DNA damage as well as cellular heterogeneity. At the cell population level, there occur two waves of apoptosis: a fast wave mediated by mitochondrial p53 within three hours postirradiation, and a slow wave mediated by nuclear p53 after eight hours postirradiation. Thus, we propose a two-step mechanism for cell fate decision. The first step is to decide whether DNA damage is severe enough to trigger apoptosis directly through the mitochondrial p53 pathway, while the second step is to determine whether the damage is fixed after cell cycle arrest. Such a mechanism may represent an efficient and reliable control mode, avoiding unnecessary death or greatly promoting the execution of apoptosis. It was also demonstrated that nuclear p53 can inhibit the pro-apoptotic activity of mitochondrial p53 by transactivating p21, and Mdm2 can facilitate apoptosis by promoting the mono-ubiquitination of p53. These results are either in good agreement with experimental observations or experimentally testable. Our work suggests that both the transcription-independent and -dependent p53 activities are indispensable for a reliable choice of cell fate and also provides clues to therapeutic manipulation of the p53 pathway in cancer treatment.

## Introduction

As a tumor suppressor, p53 lies at the hub of cellular signaling networks that are activated by various stresses including DNA damage, hypoxia and oncogene activation [1], [2]. The p53-mediated DNA damage response has been extensively studied. p53 can induce different cellular outcomes such as cell cycle arrest, senescence and apoptosis [3]. Especially, p53-mediated apoptosis is critical for suppressing tumorigenesis, and the activities of nuclear, cytoplasmic and mitochondrial p53 are all involved in apoptosis induction.

Nuclear p53 functions as a transcription factor, regulating expression of target genes. It can trigger apoptosis by transactivating pro-apoptotic genes such as Bax, Puma, Noxa and Bid and/or by repressing the expression of anti-apoptotic genes including Bcl-2, Bcl-xL and survivin [4], [5]. These lead to mitochondrial outer membrane permeabilization, cytochrome c (CytoC) release and caspase activation, and apoptosis ensues. While the nuclear p53 pathway has been recognized as the main route to apoptosis, there is now converging evidence that the transcription-independent p53 activity can induce apoptosis directly [6]–[11]. In response to death stimuli, a fraction of cellular p53 rapidly accumulates in the cytosol or mitochondria, which leads to direct activation of Bax and/or Bak and thus the initiation of apoptosis [12]–[14]. More importantly, the pro-apoptotic activity of mitochondrial p53 can come into action much faster than that of nuclear p53. It was reported that within 30 min poststimulation, p53 accumulates in mitochondria in radiosensitive organs (such as thymus and spleen), triggering a rapid wave of apoptosis that precedes the induction of pro-apoptotic p53 target genes [15]. Nevertheless, it still remains elusive how the transcription-independent and -dependent activities of p53 are orchestrated to determine cell fates after DNA damage.

The pro-apoptotic activity is a bright side of nuclear p53, by which it fulfills a tremendous tumor-suppressing function. However, convincing evidence shows that p53 also has a dark side, exhibiting powerful anti-apoptotic activity [16]. Besides the pro-apoptotic genes, nuclear p53 also transcribes several anti-apoptotic genes to counteract apoptosis. One of the main players involved is the cyclin-dependent kinase inhibitor p21, which can repress apoptosis in addition to its profound role in inhibiting proliferation. Multiple mechanisms were proposed for the anti-apoptotic activity of p21 [17]. However, it remains to be established how the anti-apoptotic activity of nuclear p53 and the pro-apoptotic activity of extranuclear p53 are functionally correlated.

Although many mathematical models were developed to explore the mechanism of p53-mediated cell fate decision [18]–[22], most of them focused on only the transcription-dependent activity of p53, and few models took into account the mitochondrial p53 pathway. Especially, the coordination between the activities of nuclear and mitochondrial p53 in the DNA damage response needs to be further elucidated.

Motivated by the above considerations, we constructed a model for the p53 network in response to DNA damage caused by 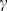-irradiation (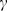
IR) and associated the network dynamics with cellular outcomes in radiosensitive tissues. By numerical simulations, we found that a reliable choice of cell fate between survival and death engages both the transcription-independent and -dependent activities of p53. We show that there appear three kinds of cellular outcomes after DNA damage: survival, apoptosis following cell cycle arrest, or immediate apoptosis. Our results indicate that both the nuclear and mitochondrial p53 pathways are important for cell fate decision and an emergence response can be triggered to kill seriously damaged cells.

## Materials and Methods

**Figure 1:**
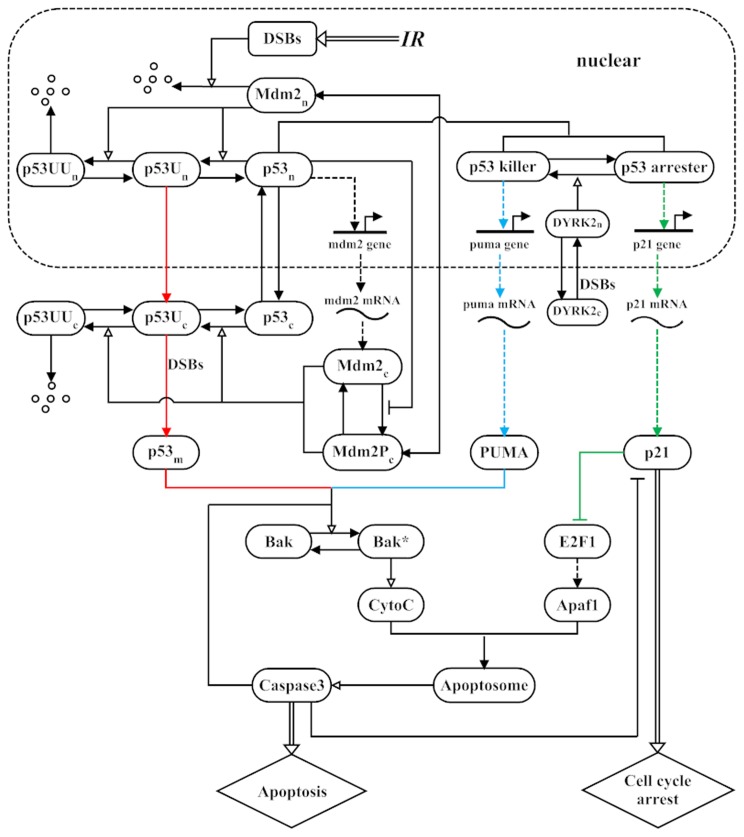
Schematic depiction of the model. Upon 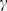-irradiation, double-strand breaks (DSBs) are generated. The degradation of Mdm2 is enhanced, while p53 is stabilized and activated. p53 is sequentially catalyzed to mono- and poly-ubiquitinated forms (p53U and p53UU) by Mdm2. Poly-ubiquitination of p53 induces its degradation by the proteasome, whereas mono-ubiquitination of p53 promotes its nuclear export and mitochondrial translocation. Based on its phosphorylation status, active nuclear p53 is distinguished between two forms, namely p53 arrester and p53 killer. They induce the expression of p21 and PUMA, respectively. DYRK2 translocates to the nucleus after DNA damage, where it promotes the conversion from p53 arrester to p53 killer. p21 indirectly suppresses E2F1 and thus Apaf1. Both mitochondrial p53 (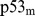) and PUMA can activate Bak. Activated Bak induces the release of CytoC from mitochondria. Once released, CytoC binds to Apaf1 and promotes the assembly of the apoptosome, activating Casp3. Activated Casp3 cleaves p21 and the inhibitors of Bak. The transcription and translation of genes are denoted by dashed lines. State transition is represented by arrow-headed solid lines, and the promotion and inhibition of state transition are separately denoted by triangle-headed and bar-headed lines. Other processes are depicted by arrow-headed double lines. Different colors are used to mark distinct p53 pathways.

We seek to reveal the mechanism for the p53-mediated cellular response to 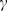
IR in radiosensitive organs of normal mice [15]. The schematic diagram of the model is depicted in Figure 1. The subnetwork of the p53-Mdm2 loop is an extension of the model in Ciliberto *et al*. [18] by incorporating the ubiquitination of cytoplasmic p53, and the subnetwork of caspase-3 (Casp3) activation is an extension of the model in Zhang *et al*. [20] by integrating the activation of Bak by both mitochondrial p53 and PUMA. Note that p53 exhibits the switch-like rather than oscillatory behavior in the experiment by Erster *et al*. [15]. Thus, the oscillatory dynamics of p53 were not considered here. The irradiation dose is denoted by 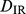.

### Simulation of the Generation and Repair of DNA Damage

Upon 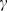
IR, double-strand breaks (DSBs) are typically induced, and DNA repair proteins are quickly recruited to break sites [23]. For a population of cells exposed to the same irradiation dose of 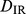, the expected total numbers of DSBs are assumed to obey a Poisson distribution with a mean of 35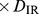 [19], [21]. Taking into account that radiation damage is more gradual and prolonged in nature, the formation and repair of DSBs are simulated simultaneously here. The production rate of DSBs is assumed to be an exponential function of time, i.e., 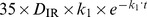 with 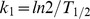 [24]. 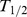 represents the time it takes for half of total DSBs to be generated. It is also assumed that there are 20 repair proteins per cell since they are much fewer than DSBs in most cases [19], [21]. The repair of DSBs can be simplified into a stochastic three-state process: a reversible binding of repair proteins and DSB into a DSB-protein complex (DSBC), and an irreversible repair process from DSBC to fixed DNA [19], [21]. The total number of unfixed DSBs and DSBCs is denoted by 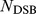. Two parallel repair pathways are included: one with fast kinetics corresponding to repair of simple DSBs, and the other with slow kinetics corresponding to repair of complex DSBs [25]. A Monte Carlo method [19], [21] was used to mimic the repair process (see Method S1 and Figure S1 for detail).

**Figure 2:**
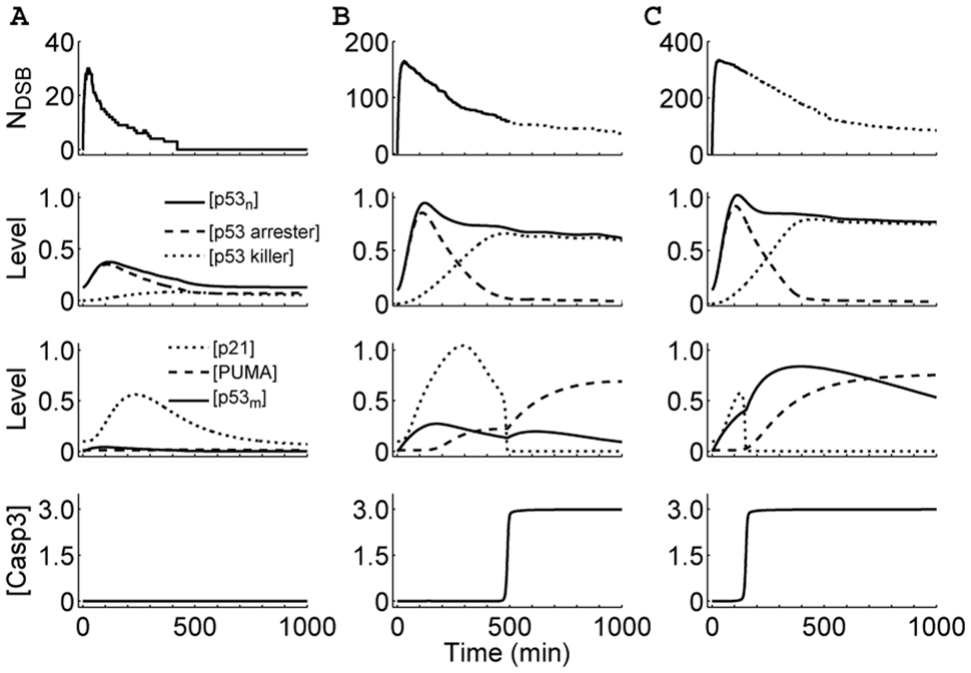
Overview of the responses of the p53 network to 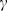-irradiation with different doses. Shown are time courses of the number of DSBs, the levels of non-ubiquitinated nuclear p53, p21, PUMA, mitochondrial p53, and Casp3 (from top to bottom) at 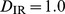 (A), 5.0 (B) or 10.0 Gy (C). The cell fate is determined by the activities of nuclear and mitochondrial p53. Depending on the extent of DNA damage, the cell may survive (A), undergo apoptosis after cell cycle arrest (B), or commit apoptosis soon after stress (C).

### Model Construction and Assumptions

DSBs are specifically detected by the ataxia-telangiectasia mutated (ATM) kinase. Upon DNA damage, ATM gets activated through auto-phosphorylation [26] and can be maintained at a relatively high level until DSBs are effectively fixed [21]. ATM transmits stress signals downstream by phosphorylating the residues in Chk2, Mdm2 and p53. Consequently, the degradation of Mdm2 is enhanced, while p53 is stabilized and activated [27]. For simplicity, we did not explicitly include ATM in the model, assuming that the ATM level can be considered constant.

**Figure 3:**
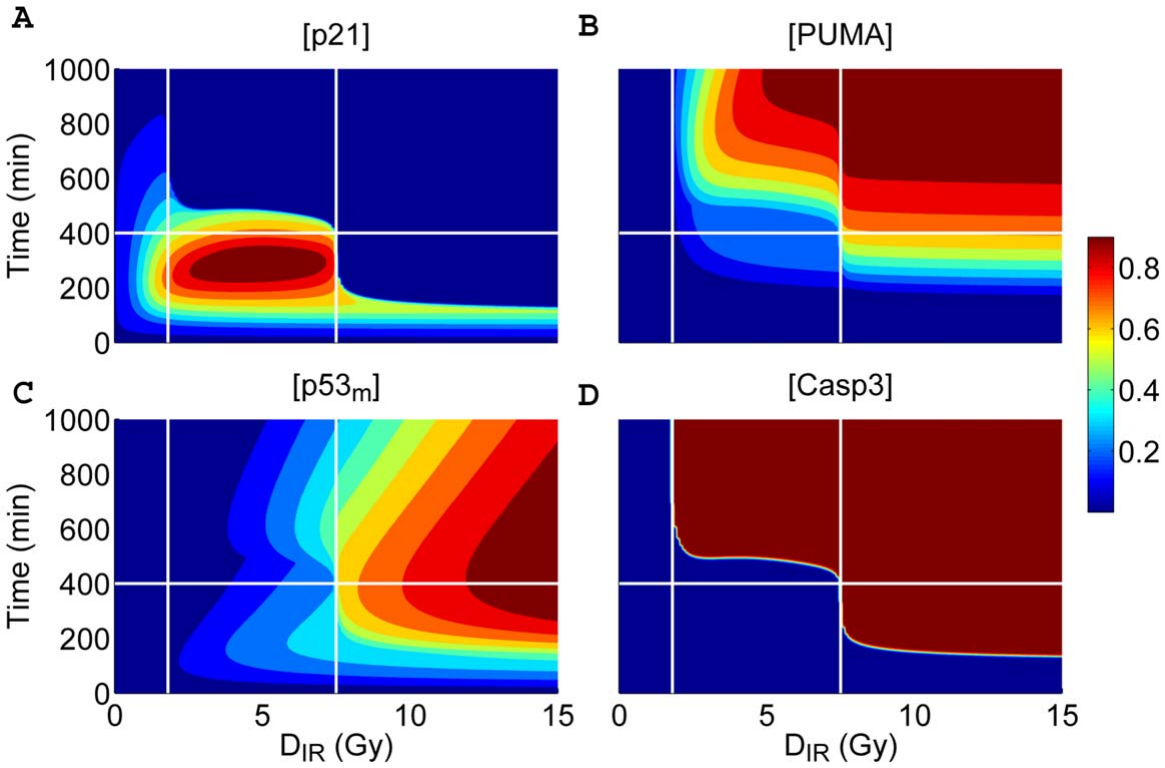
Different cellular outcomes after DNA damage. Displayed are time courses of the levels of p21 (A), PUMA (B), 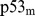 (C), and Casp3 (D) as a function of time and 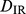. The protein levels are normalized to their respective peak values (see the color scale bar on the right). There exist three cellular outcomes: survival (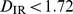 Gy), apoptosis following cell cycle (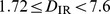 Gy), or immediate apoptosis (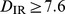 Gy).

Activated p53 induces expression of Mdm2, which in turn targets p53 for degradation, thereby enclosing a negative feedback loop. Three forms of Mdm2 are considered here, namely 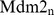 (nuclear form), 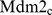 (unphosphorylated cytoplasmic form), and 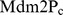 (phosphorylated cytoplasmic form). Once phosphorylated by AKT, cytoplasmic Mdm2 can translocate to the nucleus [28]. The phosphorylation of 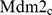 is indirectly suppressed by p53 because p53-induced PTEN inhibits the activity of AKT via PIP3 [20], [29]. The degradation rate of 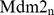 is assumed to increase with 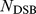 in the Michaelis-Menten form, as in Ref. [18].

**Figure 4:**
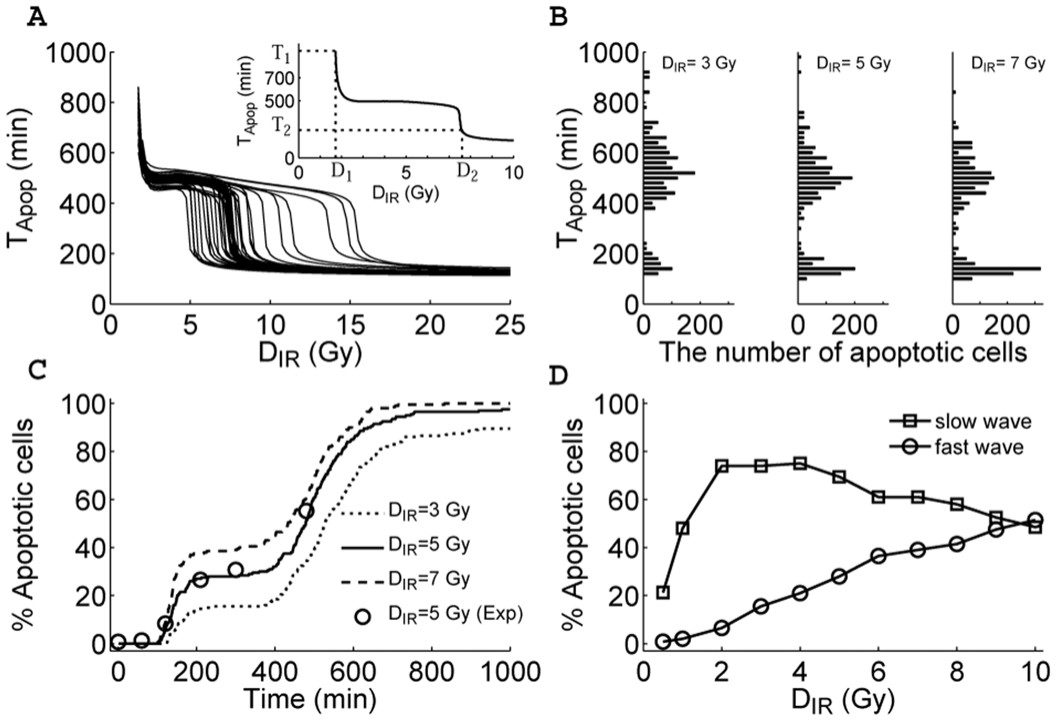
Biphasic-response patterns in apoptosis induction. The time point of Casp3 activation, 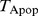, is used as a measure of timing of apoptosis. (A) Displayed are the curves of 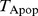 versus 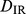 for different parameter sets where each parameter value is increased or decreased by 15% with respect to its standard value. In the inset is shown the 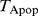 curve for the standard parameter set. The biphasic-response pattern is robust to variations in parameter values. (B) Shown are the histograms of 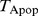 among a population of 2000 cells at 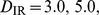 or 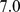 Gy (from left to right). For each cell, all parameter values are randomly chosen from 85% to 115% of their standard values, and the expected total number of DSBs obeys a Poisson distribution. The time bin is 20 min. (C) Shown is the temporal evolution of the percentage of apoptotic cells at different irradiation does by simulation (lines) or quantified experimental dada (circles) from Figure 4B in Ref. [15]. (D) Displayed is the percentage of apoptotic cells during the fast or slow wave as a function of irradiation dose.

**Figure 5:**
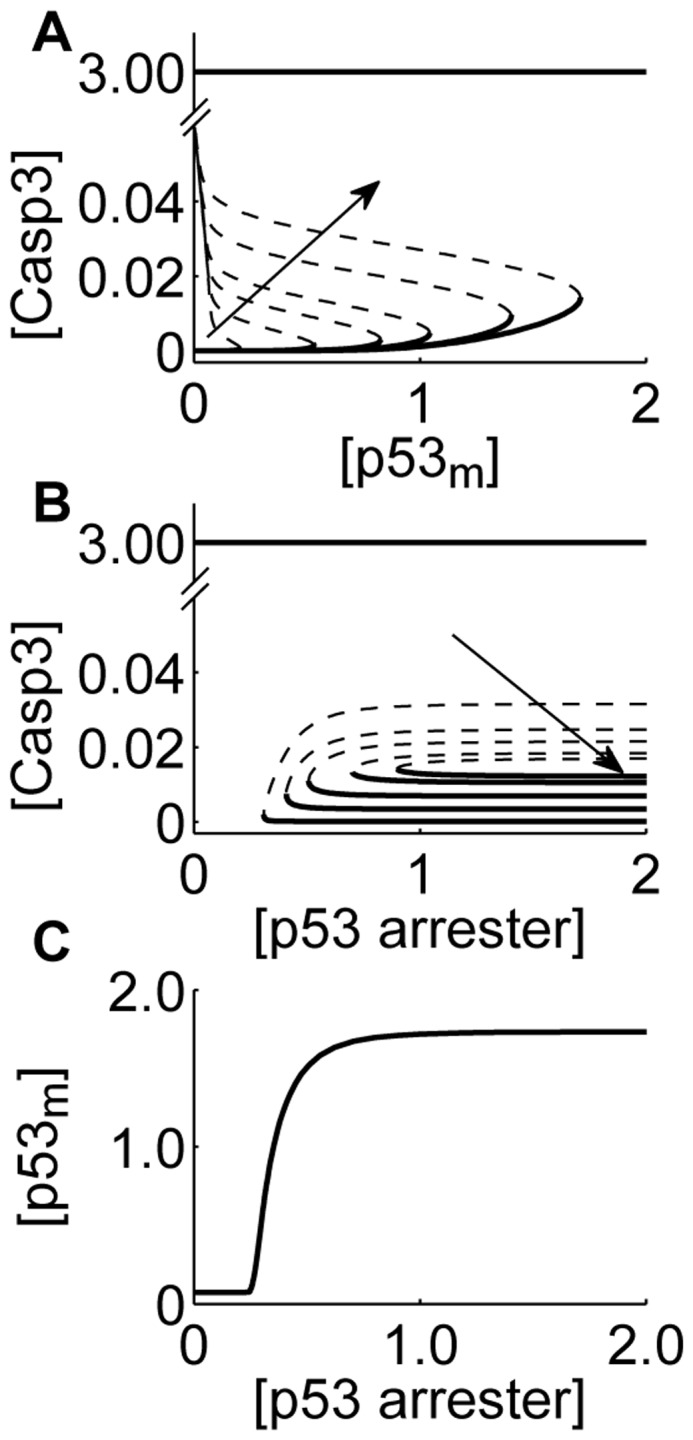
The fast wave of apoptosis determined by the competition between mitochondrial p53 and p53 arrester. (A) Displayed are the bifurcation diagrams of the Caps3 level as a function of 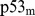 level for different levels of p53 arrester. The solid and dashed lines represent stable and unstable steady states, respectively. The line with arrow indicates the ascending order of p53 arrest. (B) Shown are the bifurcation diagrams of the Caps3 level as a function of the concentration of p53 arrester for different levels of 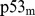. The same convention as in panel A. (C) Displayed is the two-parameter bifurcation diagram of the levels of 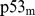 and p53 arrester. The plane is divided into two regions by the curve composed of the saddle-node bifurcation points in panels A and B.

As an E3 ubiquitin ligase for p53, Mdm2 induces its ubiquitination in the nucleus and cytoplasm [30]. Both 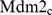 and 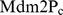 induce the ubiquitination of p53 with the same efficiency [31]. p53 is sequentially catalyzed to mono- and poly-ubiquitinated forms. Poly-ubiquitination, which requires a polymeric ubiquitin chain with at least four subunits per single lysine residue, targets p53 for efficient proteasomal degradation, whereas mono-ubiquitination, which conjugates with a ubiquitin monomer at one or multiple sites, promotes the nuclear export [30] or mitochondrial translocation of p53 [32], [33]. In each compartment (the nucleus or cytoplasm), two forms of ubiquitinated p53 are considered, namely the mono-ubiquitinated (p53U) and poly-ubiquitinated (p53UU) forms. The degradation rate of p53UU is set to be much larger than that of non-ubiquitinated p53 and p53U. The mono-ubiquitinated p53 in the nucleus (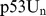) is exported into the cytoplasm. The mono-ubiquitinated p53 in the cytoplasm (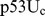) further translocates to mitochondria and then undergoes rapid deubiquitination by mitochondrial HAUSP, which is stress induced [33]. Thus, mitochondrial p53 (p53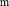) refers to the non-ubiquitinated form here.

**Figure 6:**
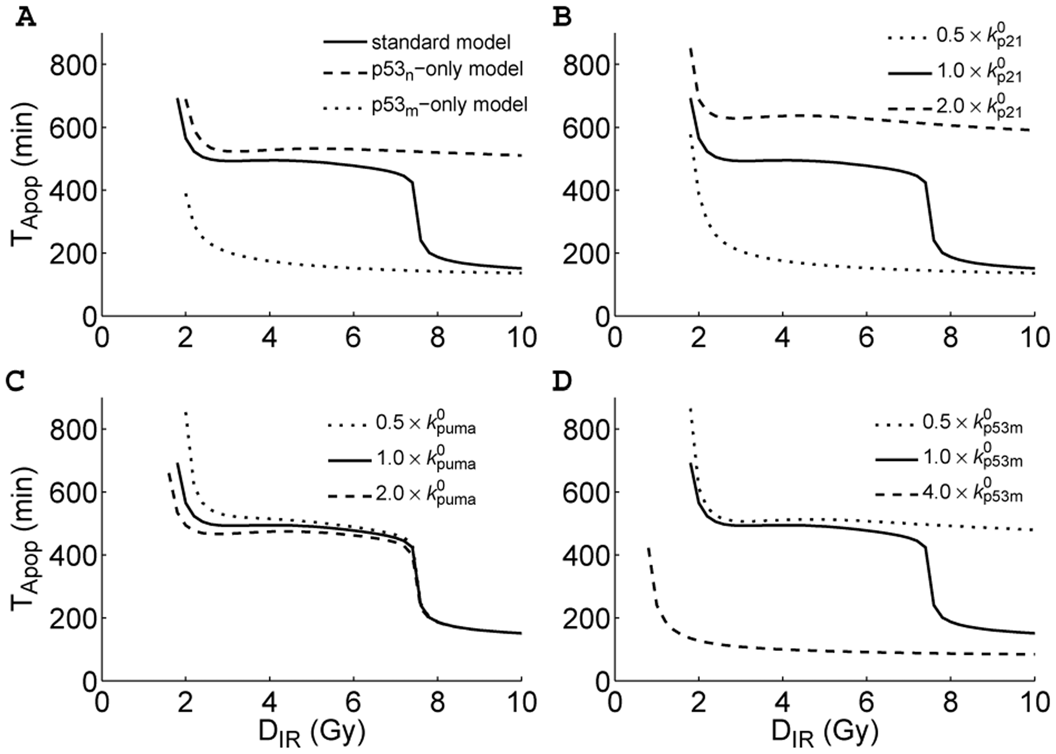
Coordination of the nuclear and mitochondrial p53 activities in apoptosis induction. (A) Shown are the 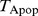 curves for three models: the standard model (solid), the 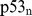-only model (dashed), and the 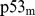-only model (dotted). The pro-apoptotic function of mitochondrial p53 is disabled in the 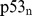-only model, whereas the transcription of genes by nuclear p53 is disrupted in the 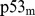-only model. (B-D) The contributions of different p53 activities to apoptosis induction. We changed the p53-dependent transcription rate of p21 (B), the p53-dependent transcription rate of PUMA (C), and the translocation rate of 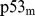 (D), respectively. Displayed are the 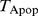 curves for different cases.

Mono-ubiquitinated nuclear p53 can still tetramerize but remain transcriptionally inactive [34], whereas non-ubiquitinated nuclear p53 (p53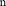) can induce gene expression. Based on its phosphorylation status, 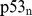 is further divided into p53 arrester and p53 killer [20], [21]. p53 arrester gets phosphorylated at Ser15/20, whereas p53 killer undergoes further phosphorylation at Ser46. Functionally, p53 arrester transactivates p21 to promote cell cycle arrest, whereas p53 killer induces PUMA to promote apoptosis. Here, the conversion from p53 arrester to p53 killer is controlled by the dual-specificity tyrosine-phosphorylation-regulated (DYRK2) kinase. DYRK2 is cytoplasmic in resting cells but translocates to the nucleus after DNA damage, where it phosphorylates p53 at Ser46 [3], [35]. The transcription rate of each gene is characterized with the Hill function. Considering the cooperativity of the tetrameric form of p53 as a transcription factor, the Hill coefficient is set to 4 [19]–[21]. The production rate of each protein is proportional to the corresponding mRNA level. Taken together, eight forms of p53 are included in the model, namely p53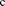 (non-ubiquitinated cytoplasmic form), p53U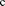, p53UU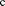, p53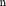 (that is divided into p53 arrester and p53 killer), p53U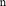, p53UU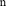, and p53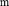.

**Figure 7:**
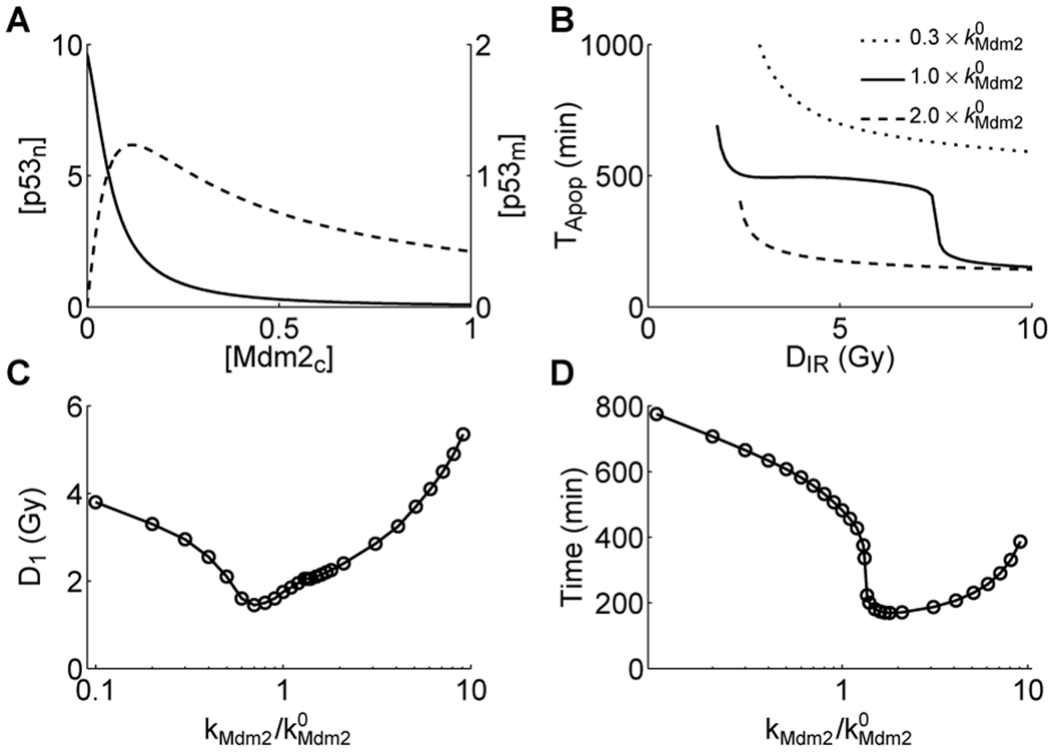
Different levels of Mdm2 have distinct effects on apoptosis induction. (A) Shown are the steady-state levels of 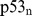 (solid) and 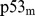 (dashed) versus the 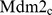 level. (B) Displayed are the 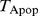 curves. The translation rate of Mdm2, 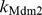, is 0.3- or 2-fold its standard value. (C) Shown is the minimal irradiation dose required for apoptosis induction, 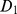, versus the ratio of the value of 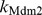 to its standard value. (D) The timing of apoptosis at 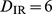 Gy versus the ratio of the value of 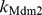 to its standard value.

Besides its principal function of inhibiting cell proliferation, p21 acts as an inhibitor of apoptosis. In our model, the anti-apoptotic function of p21 is accomplished by indirectly suppressing E2F1 and thus Apaf1 [20], which is involved in the formation of apoptosomes. Both mitochondrial p53 and PUMA can release Bak from its complex with anti-apoptotic Bcl-2 family members (such as Bcl-2, Bcl-xL and Mcl-1) and activate it [13], [14]. Activated Bak (Bak*) then leads to a loss in membrane potential of mitochondria and the release of CytoC. Once released, CytoC binds to Apaf1 and promotes the assembly of the apoptosome, which is a heptamer. This large protein complex then binds and activates Casp3. Here, persistent activation of Casp3 is considered as the indicator of apoptosis. The total level of Bak is assumed to be constant because it is constitutively located in the outer mitochondrial membrane [13], [14].

Two positive feedback loops are involved in the regulation of Casp3 activation. One is the double-negative feedback loop between p21 and Casp3: p21 indirectly inhibits Casp3 as mentioned above, whereas Casp3 cleaves and inactivates p21 [36]. The other positive feedback loop is between CytoC and Casp3: the release of CytoC leads to Casp3 activation, and Casp3 cleaves the inhibitors of Bak (such as Bcl-2 and Bcl-xL) to enhance CytoC release [37]. Interlinking the two loops constitutes a bistable apoptotic switch, which underlies a reliable cell fate decision.

### Model Simulation and Bifurcation Analysis

The dynamics of proteins and mRNA transcripts are characterized by ordinary differential equations, which are presented in Method S2. The definition of each variable and a set of standard parameter values are listed in Tables S1 and S2, respectively. The differential equations were numerically solved by using a fourth-order Runge-Kutta algorithm with a time step of 0.01 min. The initial concentration of each species was set to its stable steady-state value in the absence of DNA damage (i.e.,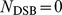), which is also listed in Table S1. The units of time and irradiation dose are minutes and Gray (Gy), respectively, while the units of other parameters ensure that the concentrations of proteins are dimensionless. The bifurcation diagrams were plotted by using Oscill8.

**Figure 8:**
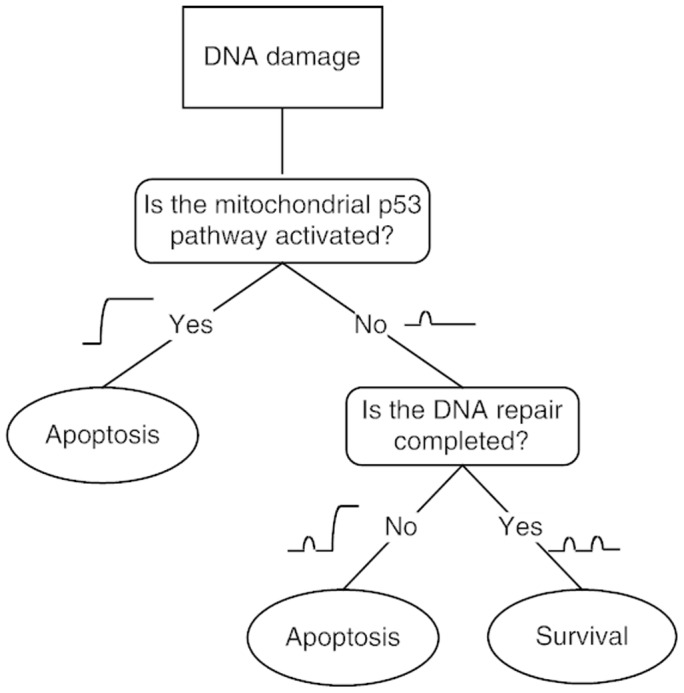
Two-step mechanism for cell fate decision. The first step is to decide whether DNA damage is severe enough to activate Casp3 through the mitochondrial p53 pathway. If yes, the apoptotic program is initiated without cell cycle arrest. Otherwise, nuclear p53 transcribes p21, which induces cell cycle arrest to allow DNA repair. Then comes the second step, determining whether the repair is completed. If yes, the cell survives; otherwise, p53 induces expression of pro-apoptotic genes, and apoptosis ensues.

## Results

### Cellular Outcomes After DNA Damage

We first explored the cellular outcome following 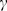
IR. Figure 2 displays the network dynamics under different irradiation conditions. Upon irradiation, DSBs are quickly generated and repair proteins are recruited to break sites. At first 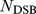 increases with time because the production rate of DSBs is larger than the repair rate. After a peak, 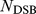 shows a decaying process with discontinuous jumps, which are due to the assumption that the step from DSBC to fixed DSB is irreversible. At 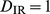 Gy, the 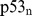 level undergoes a single low-amplitude pulse, and p53 arrester predominates over p53 killer (Figure 2A). Note that p53 arrester and p53 killer transactivate p21 and PUMA, respectively. Thus, the p21 level also exhibits a single pulse with a delay, whereas PUMA and 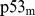 remain at basal levels. Consequently, Casp3 is kept inactive. In this case, the cell cycle is first arrested to allow time for DNA repair, and the cell recovers to normal proliferation after the damage is fixed.

At 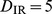 Gy, the concentration of 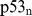 first rises fast to a relatively high level and then decreases progressively, while p53 arrester and p53 killer become dominant sequentially (Figure 2B). Thus, the p21 level first exhibits a high-amplitude pulse, while the PUMA level increases gradually. Consequently, cell cycle arrest is first induced by p21 to facilitate DNA repair. When the damage is beyond repair, enough PUMA accumulates to activate Bak. Subsequently, the release of CytoC and activation of Casp3 lead to the initiation of apoptosis around 8.3 h postirradiation. In this case, the 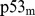 level does not change markedly during the whole response. It is worth noting that thymocytes are among the most radiosensitive cells in mammals, with a surprising speed of apoptosis induced by 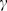
IR. It was reported that exposing cultured thymocytes to 5 Gy of 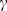
IR brings about 50% of death 8 h postirradiation [38], [39]. Therefore, the timing of apoptosis induction here is in agreement with *in vitro* findings.

At 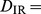
10 Gy, the concentration of 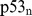 remains at a relatively high level after a transient response (Figure 2C). However, the p21 level undergoes only a short pulse with lower amplitude, whereas the 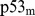 level rises remarkably soon after 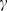
IR. Thus, there is no cell cycle arrest, and Bak is directly activated by mitochondrial p53. In this case, the cell commits apoptosis around 2.5 h postirradiation. That is, apoptosis is induced very fast at high damage levels. This is consistent with the experimental finding that a fast wave of apoptosis is triggered 2 h after 10-Gy irradiation [15]. In our simulations, Casp3 activation is all switch-like. Taken together, there appear three kinds of cellular outcomes after 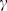
IR: survival, apoptosis following cell cycle arrest, or immediate apoptosis.

To investigate the cell fate decision systematically, we plotted the levels of p21, PUMA, 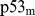 and Casp3 as a function of time and 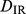 in Figure 3. To better illustrate the underlying mechanism, time courses are divided into two phases by one horizontal line (at 400 min), and the irradiation dose is designated as a low, medium or high level on three intervals separated by two vertical lines (at 1.72 and 7.6 Gy), respectively. In this way, the phase space is divided into six regions.

For 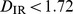 Gy, only the p21 level is significantly larger than zero in the early phase, whereas other proteins remain at basal levels. This indicates that p53 only induces p21 to arrest the cell cycle and the cell survives minor DNA damage. For 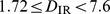 Gy, the p21 level is around its peak values over the time interval from 200 to 400 min and keeps lower in the later phase, whereas the PUMA level rises evidently during the second phase, especially at higher irradiation doses. 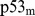 remains at a relatively low level during the whole response. Thus, it is PUMA that most promotes the activation of Bak and Casp3. Consequently, nuclear p53-induced apoptosis occurs only in the second phase. For 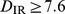 Gy, p53 quickly accumulates in mitochondria, and Bak is activated directly by 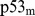. Note that the level of p21 only changes transiently because it is cleaved by Casp3. Thus, no cell cycle arrest is evoked, and apoptosis is executed rapidly. The induction of a fast apoptotic response has important implications for maintaining the integrity of the genome.

### Two Waves of Apoptosis

The above results indicate that apoptosis is induced either after cell cycle arrest or soon after irradiation, depending on the stress level. In the following, we use the time point of Casp3 activation, 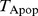, as a measure to quantitatively characterize the timing of apoptosis. For the standard parameter set, 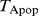 is well defined only when 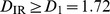 Gy (i.e., 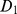 represents the minimal irradiation dose capable of inducing apoptosis; see the inset of Figure 4A). This is in agreement with the experimental observation that a significant expression of p53-targeted pro-apoptotic genes occurs in thymus and spleen when 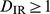 Gy [40]. With 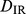 increasing, 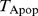 first falls fast from 934 min to 520 min and then decreases slowly until it drops sharply to be around 150 min at 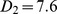 Gy. In fact, 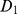 and 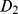 correspond to two critical points where the negative derivative of 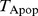 with respect to 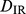 takes a local maximum (see Figure S2). The values of 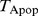 at 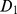 and 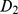 are represented by 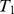 and 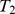, respectively. Notably, the value of 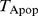 is a good manifestation of whether apoptosis is triggered quickly by mitochondrial p53 without cell cycle arrest or by nuclear p53 after cell cycle arrest.

An issue naturally arises concerning whether the above results are robust to parameter variation. To address this issue, each parameter is increased or decreased by 15% with respect to its standard value. Figure 4A displays 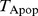 versus 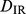 for different parameter sets. In all cases, the curves exhibit a similar trend; but 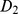 varies over a wide range, while 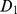 changes slightly. The relative changes in 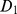, 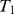, 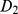 and 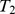 are quantified in Figure S3. Compared to the case with the standard parameter set, 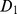 changes by less than 15% for all cases, and 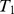 varies by 33% at most. However, 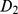 is more sensitive to some parameters, with its relative change being up to 100%, while 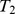 varies by 38% at most. Nevertheless, both the fast and slow apoptotic responses exist in each case.

On the other hand, it is of interest to probe the distribution of 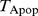 among a population of 2000 cells. As mentioned above, the expected total number of DSBs for each cell is generated from a Poisson distribution. Given cellular heterogeneity, each parameter value for every cell is randomly chosen from 85% to 115% of its standard value. Thus, the cellular responses exhibit a large variability. Notably, the histograms are all bimodal for different 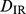: 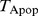 is either narrowly centered around 150 min or widely distributed around 500 min (Figure 4B). Therefore, at the population level, cells exhibit the biphasic response, and there appear two waves of apoptosis. Some cells undergo apoptosis soon after irradiation, while others may commit apoptosis at a much later time. Clearly, the first rapid wave is transcription-independent, whereas the second slow wave is transcription-dependent.

We also display how the percentage of apoptotic cells evolves temporally under different irradiation conditions in Figure 4C. There exist two time intervals where the percentage rises remarkably, corresponding to the fast and slow waves of apoptosis, respectively. It is worthy to note that our data for 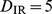 Gy are in good agreement with the experimental data [15]. Moreover, the percentage of apoptotic cells during the fast wave rises with increasing 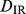, while that during the slow wave first rises and then falls with 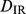 (Figure 4D). For 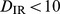 Gy, the nuclear p53-mediated apoptosis predominates over the mitochondrial p53-mediated apoptosis.

Whether apoptosis is induced quickly or not depends largely on the competition between p53 arrester and mitochondrial p53 (cf. Figure 2C). Since it is difficult to directly analyze their dynamic interactions, we can investigate the dependence of steady-state Casp3 levels on p53 arrester and 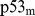, which is obtained by setting the right-hand sides of all equations in Method S2 to zero, with the levels of 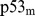 and p53 arrester being free parameters. Figure 5A shows the bifurcation diagrams of the Casp3 level as a function of 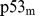 level for different levels of p53 arrester. The Casp3 level can switch from the lower to the upper state when the 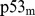 level crosses the saddle-node bifurcation point. Once Casp3 is settled into the upper state, it stays there independently of 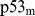. This is responsible for the irreversible induction of apoptosis. The threshold for switching on Casp3 increases with the level of p53 arrester. That is, when the level of p53 arrester becomes larger, more accumulation of 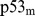 is needed to turn on the Casp3 switch. On the other hand, Casp3 is switched on only with low levels of p53 arrester, and improving 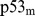 levels contributes to the switching (Figure 5B).

In the parameter space spanned by the levels of 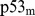 and p53 arrester, two regions are separated by the curve composed of the above bifurcation points (Figure 5C). The top left corresponds to Casp3 activation, while the other corresponds to Casp3 inactivation. This indeed indicates that an amount of mitochondrial p53 can lead to Casp3 activation when the level of p53 arrester is controlled within an appropriate range. Therefore, whether fast apoptosis can be induced rests on whether mitochondrial p53 predominates over p53 arrester.

### Coordination of Nuclear and Mitochondrial p53 Activities

Our results suggest that both the transcription-dependent and -independent activities of p53 are indispensable for making a reliable cell-fate decision. To see this clearly, we constructed two variant models: the 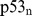-only model where the pro-apoptotic function of mitochondrial p53 is disabled (i.e., 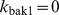), and the 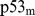-only model where the transcriptional activity of nuclear p53 is disrupted (i.e., 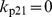, 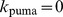 and 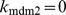). Such assumptions are based on the reports that the pro-apoptotic activity of mitochondrial p53 can be inhibited by Pifithrin-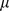 [41] and the role of nuclear p53 as a transcription factor can be blocked by Pifithrin-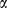 [42].

As the value of 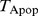 is a good reflection of the mechanism for Casp3 activation, we plotted 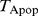 versus 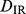 for three models (with the standard parameter set) in Figure 6A. In the 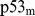-only model, 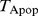 drops from 390 min at 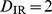 Gy to a minimum of less than 140 min. That is, when p21, PUMA and Mdm2 are expressed only at basal levels, apoptosis always ensues soon after stress. It is noted that although p53-dependent expression of Mdm2 is inhibited, its basal expression generates low level of Mdm2, which promotes the mono-ubiquitination and mitochondrial translocation of p53 (see Figure S4). Moreover, because the inhibition of apoptosis by p21 is released, it is easier to trigger the rapid apoptosis by mitochondrial p53. Furthermore, decreasing the basal transcription rate of Mdm2 may enhance p53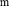-induced apoptosis (see the red lines in Figure S4), and this point will be discussed later. These results are consistent with the experimental observations that disrupting the transcriptional activity of p53 greatly enhances rather than inhibits apoptosis induction [43]–[45]. This also suggests that combining anticancer drugs (like Nutlin-3, which can upregulate p53 levels) with inhibitors of nuclear p53 (like Pifithrin-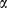) may be a promising way to improve the therapy efficacy.

In the 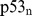-only model, however, 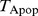 drops from 690 min at 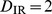 Gy to 510 min at 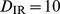 Gy. Thus, apoptosis is always evoked slowly by nuclear p53 alone, even at high damage levels. That is, blocking the extra-nuclear activity of p53 greatly delays the initiation of apoptosis. Therefore, when the function of nuclear p53 is interrupted, inappropriate cell death may be evoked; when the function of mitochondrial p53 is disrupted, seriously damaged cells cannot be destroyed timely. By contrast, the coordination of both p53 activities can ensure a balance between cell survival and death.

We further explored the influence of different p53 activities on apoptosis induction. As mentioned above, nuclear p53 also has an anti-apoptotic role via transcribing p21. To see this clearly, we changed the p53-dependent transcription rate of p21, 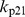, and plotted the 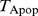 curves in Figure 6B. When 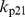 is doubled or halved, 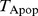 decreases with increasing 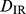 along the upper and the lower branch, respectively. That is, the biphasic behavior disappears. When p21 is transcribed faster, it takes a longer time to induce apoptosis because p21 exerts a stronger suppression of E2F1 activity. By contrast, when p21 is transcribed more slowly, it takes less time to trigger apoptosis. Therefore, upregulating p21 expression tends to repress the induction of apoptosis.

On the other hand, doubling or halving the p53-dependent transcription rate of PUMA (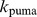) has a much smaller effect on the 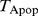 curve (Figure 6C). When 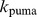 is increased from 0 to two-fold its default value, 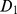 dwindles gradually from 3.4 to 1.5 Gy, but 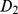 varies slightly (Figure S5). Thus, the pro-apoptotic activity of nuclear p53 regulates the slow wave of apoptosis but has little effect on the nature of the biphasic response.

As shown above, the fast wave of apoptosis is triggered by mitochondrial p53. Enhancing the translocation rate of 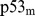 can always facilitate the induction of apoptosis because Casp3 is activated more quickly, escaping the repression of p21 (Figure 6D). By contrast, weakening the translocation rate of 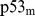 may delay or abolish the fast wave of apoptosis. Thus, the activity of mitochondrial p53 should be sufficiently strong so as to quickly remove severely damaged cells. Taken together, both the nuclear and mitochondrial p53 pathways are required to evoke an optimal DNA damage response.

### Two Faces of Mdm2: Inhibiting or Promoting Apoptosis Induction

It is widely accepted that Mdm2 is an oncoprotein because it is the major E3 ubiquitin ligase for p53. It is known that higher levels of Mdm2 promote poly-ubiquitination of p53, while lower levels of Mdm2 promote its mono-ubiquitination [30]. Mono-ubiquitinated nuclear p53 can be exported to the cytoplasm and further translocate to mitochondria upon stress. Thus, it is necessary to investigate the influence of Mdm2 on the mitochondrial p53 pathway.

We first analyzed the dependence of the steady-state levels of 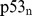 and 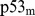 on 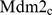. The 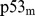 level first rises and then falls with increasing the 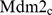 level, whereas the 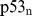 level drops monotonically (Figure 7A). This is consistent with the experimental observation of how the ubiquitination of p53 depends on Mdm2 [30]. Thus, there exists some possibility that mitochondrial p53-mediated apoptosis can be facilitated by improving Mdm2 levels over some range. That is, Mdm2 may have a tumor-suppressing function via promoting the mono-ubiquitination of p53. This is in contrast with the classical view that Mdm2 is only an oncoprotein.

To address this possibility, we compared the 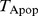 curves between the cases with different translation rates of Mdm2 (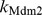) in Figure 7B. Clearly, when 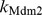 is 0.3-fold its standard value, the fast wave of apoptosis disappears. Moreover, 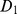 rises evidently, and it takes more time to trigger apoptosis. That is, downregulating the expression of Mdm2 tends to inhibit apoptosis. By contrast, when 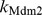 is two-fold its standard value, it takes much less time to induce apoptosis but 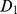 becomes larger. Thus, upregulating the expression of Mdm2 has a more complicated effect on apoptosis induction; whether Mdm2 promotes or inhibits apoptosis also depends on the extent of DNA damage, which affects the degradation rate of Mdm2.

Furthermore, we plotted the minimal irradiation dose required for apoptosis induction versus the ratio of the value of 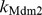 to its standard value in Figure 7C. 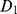 first decreases and then rises with increasing the ratio. Moreover, the time taken to initiate apoptosis at 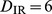 Gy also first decreases and then rises with increasing the ratio (Figure 7D). Thus, Mdm2 with appropriate levels may act as a tumor suppressor. That is, Mdm2 has two faces; under certain conditions, it can promote apoptosis by reducing the minimal irradiation dose for apoptosis induction and/or speeding up apoptosis initiation. Therefore, changing Mdm2 levels may switch the role of Mdm2 between a tumor suppressor and an oncoprotein. Accordingly, enhancing the pro-apoptotic activity of mono-ubiquitinated p53 by targeting the p53-Mdm2 interaction could be a potential therapeutic strategy in cancer treatment.

## Discussion

In the present work, we developed a model of the p53 signaling network in radiosensitive tissues to clarify how the cell fate decision is governed by the nuclear and mitochondrial p53 pathways. We found that depending on the extent of DNA damage, there exist three kinds of cellular outcomes: 1) the cell survives, 2) the cell commits apoptosis after cell cycle arrest, or 3) the cell undergoes apoptosis soon after irradiation. At the population level, apoptosis appears in two waves: the fast wave mediated by the mitochondrial p53 pathway and the slow wave by the nuclear p53 pathway. Therefore, we propose a two-step mechanism for cell fate decision (Figure 8). The first step is to decide whether DNA damage is severe enough to activate Casp3 directly through the mitochondrial p53 pathway. If yes, the apoptotic program is initiated, and those cells are quickly eliminated with little time for DNA repair. Otherwise, nuclear p53 transcribes p21, which induces cell cycle arrest to allow DNA repair. The second step then determines whether the repair is beyond repair. If yes, nuclear p53 induces expression of pro-apoptotic genes, and apoptosis ensues; otherwise, the cell survives.

Such a two-step mechanism may represent an optimal control mode of the DNA damage response in radiosensitive tissues. Most previous models assumed that cells always first arrest the cell cycle to fix DNA damage no matter how severe it is [20], [21]. This seems not so favorable for three reasons. First, it is a waste of energy to try to repair seriously damaged cells because they are destined for death. Second, it is dangerous to repair severe DNA damage since this could lead to genomic instability. Third, an emergency-response mechanism is needed to eliminate seriously damaged cells timely. By contrast, this two-step mechanism provides an efficient and reliable control.

We have emphasized that the fast apoptosis is induced by the mitochondrial p53 pathway. In fact, cytoplasmic p53 also has a pro-apoptotic function when it is released from sequestration by Bcl-2 or Bcl-xL, which is promoted by PUMA. Nevertheless, PUMA expression is controlled by nuclear p53 [46], [47]. Thus, this endogenous pro-apoptotic function of cytoplasmic p53 still depends on nuclear p53. That is, the cytoplasmic p53 pathway cannot be qualified for triggering an emergency response, which instead engages the mitochondrial p53 pathway.

We demonstrated that Mdm2 has two faces in apoptosis induction. Rather than simply repress apoptosis, Mdm2 with moderate levels can promote apoptosis by facilitating or/and speeding up apoptosis induction. Indeed, converging evidence supports the remarkable possibility that Mdm2 has a tumor-suppressing function within the appropriate context [48]. For example, Mdm2 can suppress cell proliferation [49], [50] or lead to instability of mutant p53 in tumor [51]. Here, we found that Mdm2 can promote apoptosis via mono-ubiquitination of p53. This may be exploited to develop Mdm2-based cancer therapy.

The p53 dynamics in the DNA damage response are actually cell-, tissue- and stress-specific. For example, oscillatory behaviors of p53 have been reported both experimentally and theoretically [19], [21], [52]–[55]. Positive and negative feedback loops have been identified underlying p53 oscillations [22]. However, there is no evidence supporting oscillatory p53 behavior in the experiment by Erster *et al*. [15]. Thus, the oscillatory dynamics of p53 were not considered in our model. But it would be interesting to check how the coordination of nuclear and mitochondrial p53 activities is affected by their response modes.

Our results suggest that a reliable cell fate decision involves the coordination between the nuclear and mitochondrial p53 pathways, which may be exploited in cancer treatment. The cell cycle arrest and slow wave of apoptosis can be induced by nuclear p53, whereas mitochondrial p53 can trigger an emergency response at high damage levels. Disrupting cell cycle arrest can greatly enhance apoptosis induction. Thus, a possible strategy for cancer therapy is to combine anticancer drugs with inhibitors of nuclear p53. This could lower the dosage of drugs and shorten the medication time. Therefore, manipulating both the nuclear and mitochondrial p53 pathways may be a promising way to improve the therapy efficacy.

## Supporting Information

Figure S1 **The two-lesion kinetic model of DNA repair.** Two parallel repair pathways are considered: one with fast kinetics corresponding to repair of simple DSBs, and the other with slow kinetics corresponding to repair of complex DSBs. For both pathways, the repair of DNA damage is simplified into a three-state process: a reversible binding of repair proteins and DSB (‘D’) into DSBC (‘C’), followed by an irreversible repair process from DSBC to fixed DNA (‘F’). Subscripts ‘1’ and ‘2’ are used to distinguish fast kinetics from slow kinetics.
(TIFF)

Figure S2
**Definitions of the minimal irradiation dose capable of inducing apoptosis (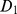) and that capable of inducing fast apoptosis (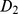).** Shown is the negative derivative of 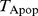 with respect to 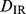, which is defined as 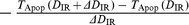. The derivative takes a local maximum at 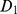 and 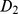.(TIFF)

Figure S3
**Parameter sensitivity analysis of 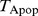.** Here, each parameter is increased (pentagon) or decreased (circle) by 15% with respect to its standard value. Compared to the case with the standard parameter set, the relative changes in 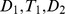 and 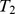 are separately quantified in panels A–D.(TIFF)

Figure S4
**Temporal evolution of protein levels in the p53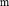-only model at 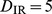 Gy.** Shown are the dynamics of the levels of Mdm2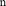 (A), p53U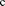 (B), p53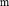 (C) and Casp3 (D) for different basal levels of Mdm2.(TIFF)

Figure S5
**The dependence of 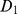 and 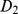 on the p53-dependent transcription rate of puma, 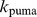. 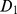 and 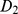 versus the ratio of the value of 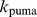 to its standard value are shown in A and B, respectively.**
(TIFF)

Method S1 **Simulation method for the repair of DNA damage.**
(PDF)

Method S2 **Ordinary differential equations for the model.**
(PDF)

Table S1 **Description and initial values of the model variables.**
(PDF)

Table S2 **Description and values of the model parameters.**
(PDF)
