# Supplementary figures and images for: A Two-Step Mechanism for Cell Fate Decision by Coordination of Nuclear and Mitochondrial p53 Activities
Source: PLoS One. 2012 Jun 5;7(6):e38164. doi: 10.1371/journal.pone.0038164 (PMC3367989; doi:10.1371/journal.pone.0038164)

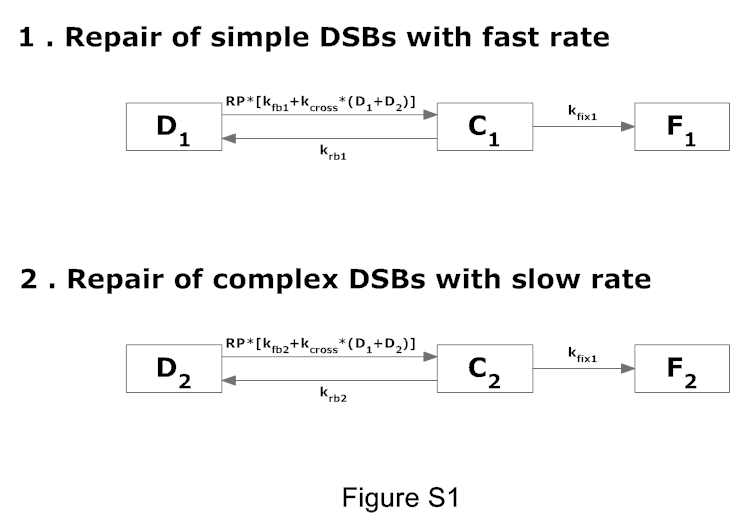

Supplement: Figure S1 — The two-lesion kinetic model of DNA repair. Two parallel repair pathways are considered: one with fast kinetics corresponding to repair of simple DSBs, and the other with slow kinetics corresponding to repair of complex DSBs. For both pathways, the repair of DNA damage is simplified into a three-state process: a reversible binding of repair proteins and DSB (‘D’) into DSBC (‘C’), followed by an irreversible repair process from DSBC to fixed DNA (‘F’). Subscripts ‘1’ and ‘2’ are used to distinguish fast kinetics from slow kinetics. (TIFF) [file pone.0038164.s001.tiff]

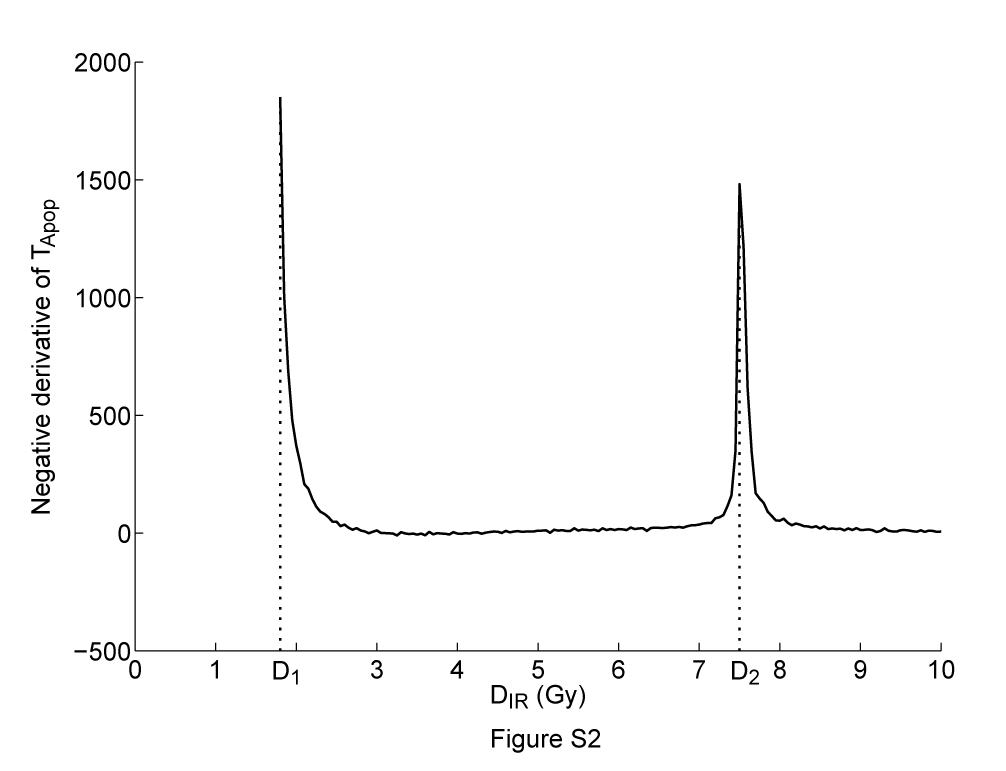

Supplement: Figure S2 — Definitions of the minimal irradiation dose capable of inducing apoptosis () and that capable of inducing fast apoptosis (). Shown is the negative derivative of with respect to , which is defined as . The derivative takes a local maximum at and . (TIFF) [file pone.0038164.s002.tiff]

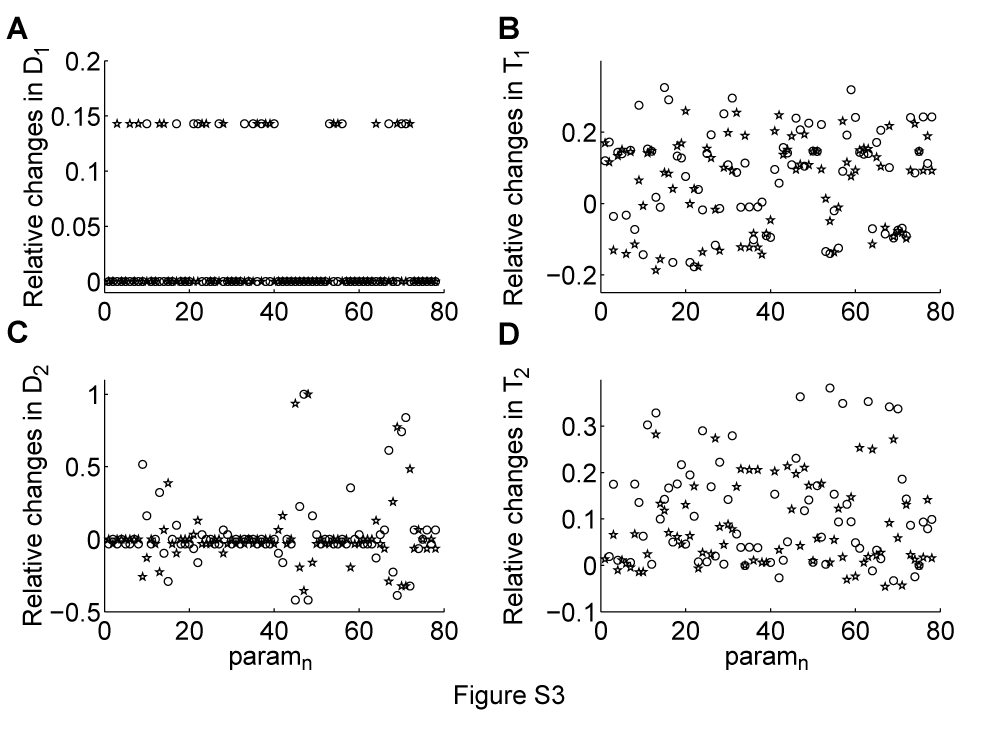

Supplement: Figure S3 — Parameter sensitivity analysis of . Here, each parameter is increased (pentagon) or decreased (circle) by 15% with respect to its standard value. Compared to the case with the standard parameter set, the relative changes in and are separately quantified in panels A–D. (TIFF) [file pone.0038164.s003.tiff]

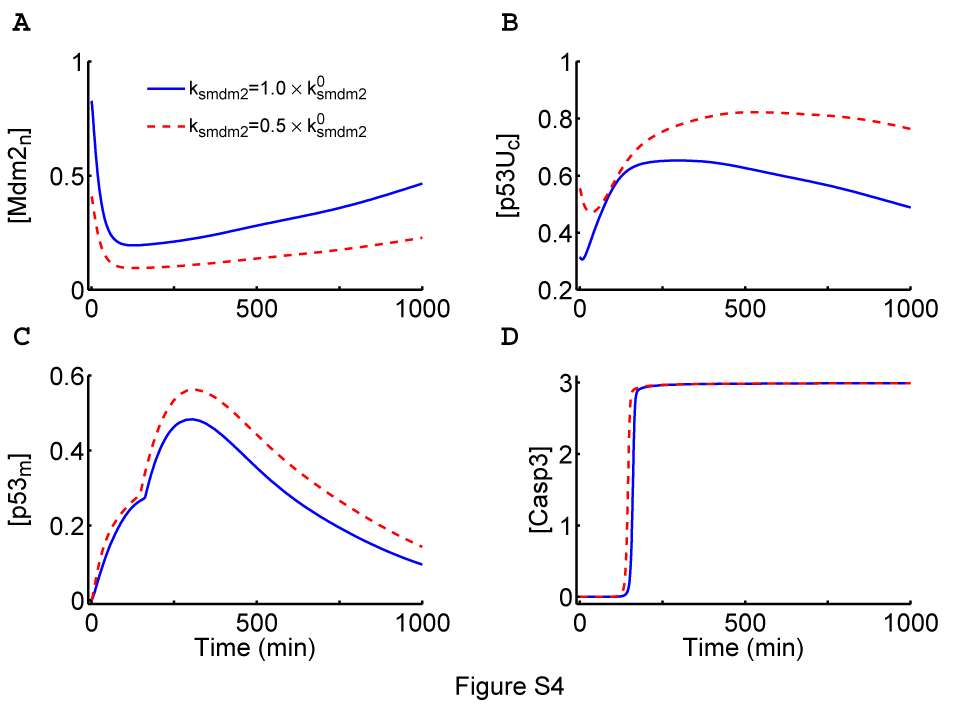

Supplement: Figure S4 — Temporal evolution of protein levels in the p53-only model at Gy. Shown are the dynamics of the levels of Mdm2 (A), p53U (B), p53 (C) and Casp3 (D) for different basal levels of Mdm2. (TIFF) [file pone.0038164.s004.tiff]

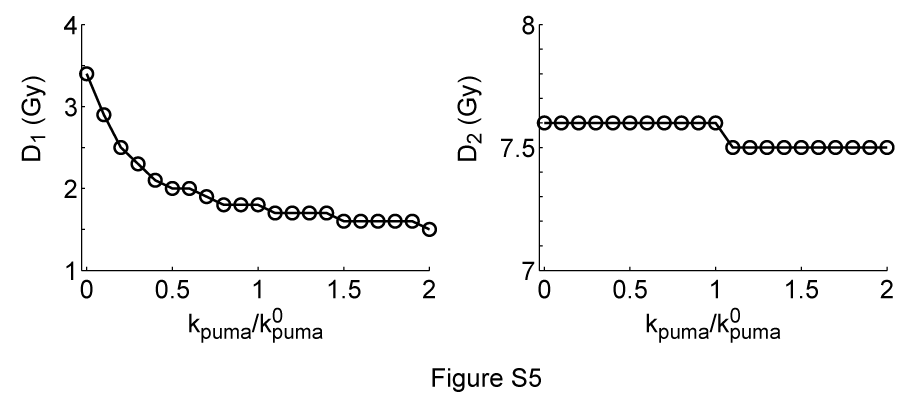

Supplement: Figure S5 — The dependence of and on the p53-dependent transcription rate of puma, . and versus the ratio of the value of to its standard value are shown in A and B, respectively. (TIFF) [file pone.0038164.s005.tiff]
