# Supplementary material for: A Two-Step Mechanism for Cell Fate Decision by Coordination of Nuclear and Mitochondrial p53 Activities
Source: PLoS One. 2012 Jun 5;7(6):e38164. doi: 10.1371/journal.pone.0038164 (PMC3367989; doi:10.1371/journal.pone.0038164)
Supplement: Method S1 — Simulation method for the repair of DNA damage. (PDF) [file pone.0038164.s006.pdf]

SUPPORTING MATERIAL

A Two-Step Mechanism for Cell Fate Decision by Coordination of Nuclear and Mitochondrial p53 Activities

Xiao-Jun Tian, Feng Liu, Xiao-Peng Zhang, Jun Li, and Wei Wang

In this work, we aim to reveal the mechanism for the p53-mediated cellular response to γ-irradiation (γIR) in radiosensitive organs (such as thymus, spleen and testis) of normal mice [1]. We focus on the activities of nuclear and mitochondrial p53. Nuclear p53 serves as a transcription factor, transactivating either p21 for anti-apoptosis or puma for pro-apoptosis, whereas mitochondrial p53 has a direct pro-apoptotic activity. Remarkably, the activity of mitochondrial p53 comes into action much faster than that of nuclear p53. In the following, we present the details of the model.

Method S1: Simulation method for the repair of DNA damage

When living cells are exposed to ionizing radiation (IR), a large variety of DNA lesions are generated. Among them, the most dangerous is double-strand break (DSB), with both strands in the double helix severed. In mammalian cells, there exist two major repair pathways: nonhomologous end joining (NHEJ) and homologous recombination (HR) [2, 3]. HR mainly contributes to DSB repair in the S phase, whereas NHEJ is the predominant pathway of DSB repair, especially active in the G1 phase [4]. Here, NHEJ is taken as the pathway of DSB repair. Although it is believed that p53 has a role in DNA repair, the detailed effect of p53 on DNA repair is complicated and controversial. Thus, the effect of p53 on DNA repair is not considered here.

Owing to stochasticity in the generation and repair of DSBs, a Monte Carlo method is used to simulate the repair process [5, 6]. The irradiation dose is in units of Gy and is denoted by D_{IR}. In simulations, the expected total number of DSBs in each cell is generated from a Poisson distribution with a mean of $35\times D_{\mathrm{IR}}$, and the total number of repair proteins is assumed to be 20 per cell. Taking into account that radiation damage is more gradual and prolonged in nature, the formation and repair of DSBs are simulated simultaneously here. The production rate of DSBs is assumed to be an exponential function of time, i.e., $35 \cdot D_{\mathrm{IR}} \cdot k_1 \cdot e^{-k_1 \cdot t}$ with $k_1 = ln2/T_{1/2}$ [7]. $T_{1/2}$ represents the time it takes for half of total DSBs to be generated. The repair of DSBs can

be simplified into a stochastic three-state process: a reversible binding of repair proteins and DSB ('D') into a DSB-protein complex (DSBC) ('C'), followed by an irreversible repair process from DSBC to fixed DNA ('F') (see Fig. S1). Two parallel repair pathways are considered: one with fast kinetics corresponding to repair of simple DSBs, and the other with slow kinetics corresponding to repair of complex DSBs [8]. We use subscripts '1' and '2' to distinguish fast kinetics from slow kinetics. The Monte Carlo algorithm for the repair dynamics is based on the transition probabilities between two neighboring states:

$$P_{\mathrm{D_1 \to C_1}} = RP[k_{\mathrm{fb1}} + k_{\mathrm{cross}}(N_{\mathrm{D1}} + N_{\mathrm{D2}})]\Delta t \quad (1)$$

$$P_{\mathrm{D_2 \to C_2}} = RP[k_{\mathrm{fb2}} + k_{\mathrm{cross}}(N_{\mathrm{D1}} + N_{\mathrm{D2}})]\Delta t \quad (2)$$

$$P_{\mathrm{C_1 \to D_1}} = k_{\mathrm{rb1}}\Delta t \quad (3)$$

$$P_{\mathrm{C_2 \to D_2}} = k_{\mathrm{rb2}}\Delta t \quad (4)$$

$$P_{\mathrm{C_1 \to F_1}} = k_{\mathrm{fix1}}\Delta t \quad (5)$$

$$P_{\mathrm{C_2 \to F_2}} = k_{\mathrm{fix2}}\Delta t. \quad (6)$$

All the ratios of the rates for the fast kinetics to those for the slow kinetics are chosen to be close to 10 (i.e., $k_{\mathrm{fb1}}/k_{\mathrm{fb2}} = k_{\mathrm{rb1}}/k_{\mathrm{rb2}} = k_{\mathrm{fix1}}/k_{\mathrm{fix2}} = 10$), which is based on the experimental data that these ratios are about 6-40 [8]. It is also assumed that the binding of repair proteins to DSBs is much faster than other processes such as dissociation of repair proteins and the repair process. In simulation, the fraction of simple DSBs is set to 0.7. The total number of unrepaired DSBs and DSBCs is denoted by N_{DSB}. In addition to the stochasticity in the generation and repair of DSBs, parameter values can be different between cells to account for the cell-to-cell variability.

The Monte Carlo algorithm for the dynamics of DSB repair at D_{IR} during the time interval from 0 to T_{max} is presented as follows.

1. Set the parameters for the cell

   Each parameter value is randomly chosen from 85% to 115% of its standard value.

2. Initialization

   Set t = 0. The initial number of DSBs is $N_{\mathrm{DSBT}}(0) = 0$, and the initial values for simple and complex DSB repair are taken as $N_{\mathrm{D_1}}(0) = 0$ and $N_{\mathrm{D_2}}(0) = 0$, respectively. The number of free repair proteins is $N_{\mathrm{RP}}(0) = 20$. $N_{\mathrm{C_1}}(0) = N_{\mathrm{C_2}}(0) = N_{\mathrm{F_1}}(0) = N_{\mathrm{F_2}}(0) = 0$.

3. Increment time

   Set t = t + Δt.

4. Generation of DSBs.

   The expected number of DSBs, N_{DSBT}(t), is set to the nearest integer less than or equal to $35D_{\mathrm{IR}}(1 - e^{-t \cdot k_1})$. The expected numbers of simple and complex DSBs are taken as $N_{\mathrm{D_{1T}}}$(t)=0.7N_{DSBT} (t) and $N_{\mathrm{D_{2T}}}$(t)= 0.3N_{DSBT}(t), respectively. The numbers of simple and complex DSBs in state 'D' are $N_{\mathrm{D_1}}(t) = N_{\mathrm{D_{1T}}}(t) - N_{\mathrm{C_1}}(t - \Delta t) - N_{\mathrm{F_1}}(t - \Delta t)$ and $N_{\mathrm{D_2}}(t) = N_{\mathrm{D_{2T}}}(t) - N_{\mathrm{C_2}}(t - \Delta t) - N_{\mathrm{F_2}}(t - \Delta t)$, respectively.

5. Update the states for each break site controlled by fast repair

   For each damage locus i (with $1 \leq i \leq N_{\mathrm{D_{1T}}}(t)$), a random number r is generated from a uniform distribution between 0 and 1. If the damage at locus i is in state 'D' and $N_{\mathrm{RP}} > 0$, a transition to state 'C' occurs if $0 \leq r < P_{\mathrm{D_1 \to C_1}}$, whereas it stays in state 'D' if $P_{\mathrm{D_1 \to C_1}} \leq r \leq 1$. If the damage is in state 'C', a transition to state 'D' occurs if $0 \leq r < P_{\mathrm{C_1 \to D_1}}$, or a transition to state 'F' occurs if $P_{\mathrm{C_1 \to D_1}} \leq r \leq P_{\mathrm{C_1 \to D_1}} + P_{\mathrm{C_1 \to F_1}}$. If the damage is in state 'F', it always stays there (i.e., state 'F' is absorbing). Set $N_{\mathrm{RP}} = N_{\mathrm{RP}} + 1$ if the transition from state 'C' to 'D' or from state 'C' to 'F' occurs; set $N_{\mathrm{RP}} = N_{\mathrm{RP}} - 1$ if the transition from state 'D' to 'C' occurs; otherwise N_{RP} remains the same. After the last break site has been updated, set the numbers of fast repaired breaks at time t in states 'D', 'C', and 'F' to $N_{\mathrm{D_1}}$, $N_{\mathrm{C_1}}$ and $N_{\mathrm{F_1}}$, respectively.

6. Update the states for each break site controlled by slow repair

   For each damage locus i (with $1 \leq i \leq N_{\mathrm{D_{2T}}}(0)$), a random number r is generated from a uniform distribution between 0 and 1. If the damage at locus i is in state 'D' and $N_{\mathrm{RP}} > 0$, a transition to state 'C' occurs if $0 \leq r < P_{\mathrm{D_2 \to C_2}}$, while it stays in state 'D' if $P_{\mathrm{D_2 \to C_2}} \leq r \leq 1$. If the damage is in state 'C', a transition to state 'D' occurs if $0 \leq r < P_{\mathrm{C_2 \to D_2}}$, or a transition to state 'F' occurs if $P_{\mathrm{C_2 \to D_2}} \leq r \leq P_{\mathrm{C_2 \to D_2}} + P_{\mathrm{C_2 \to F_2}}$. If the damage is in state 'F', it always stays there (i.e., state 'F' is absorbing). Set $N_{\mathrm{RP}} = N_{\mathrm{RP}} + 1$ if the transition from state 'C' to 'D' or from state 'C' to 'F' occurs; set $N_{\mathrm{RP}} = N_{\mathrm{RP}} - 1$ if the transition from state 'D' to 'C' occurs; otherwise N_{RP} remains the same. After the last damage site has been updated, set the numbers of slow repaired breaks at time t in states 'D', 'C', and 'F' to be $N_{\mathrm{D_2}}$, $N_{\mathrm{C_2}}$ and $N_{\mathrm{F_2}}$, respectively.

7. Let $N_{\mathrm{D}}(t) = N_{\mathrm{D_1}}(t) + N_{\mathrm{D_2}}(t)$, $N_{\mathrm{C}}(t) = N_{\mathrm{C_1}}(t) + N_{\mathrm{C_2}}(t)$, $N_{\mathrm{F}}(t) = N_{\mathrm{F_1}}(t) + N_{\mathrm{F_2}}(t)$

8. Repeat steps 3-7 until all of the DSBs are effectively repaired (i.e., $N_{\mathrm{D}}(t) + N_{\mathrm{C}}(t) < 2$) or $t = T_{\mathrm{max}}$.

[1] Erster S, Mihara M, Kim RH, Petrenko O, Moll UM (2004) In vivo mitochondrial p53 translocation triggers a rapid first wave of cell death in response to DNA damage that Can precede p53 target gene activation. Mol Cell Biol 24:6728-41.

[2] Sengupta S, Harris CC (2005) p53: traffic cop at the crossroads of DNA repair and recombination. Nat Rev Mol Cell Biol 6:44-55.

[3] Gatz SA, Wiesmuller L (2006) p53 in recombination and repair. Cell Death Differ 13:1003-16.

[4] Burma S, Chen BP, Chen DJ (2006) Role of non-homologous end joining (NHEJ) in maintaining genomic integrity. DNA Repair 5:1042-48.

[5] Ma L, Wagner J, Rice JJ, Hu W, Levine AJ, Stolovitzky GA (2005) A plausible mode for the digital response of p53 to DNA damage. Proc Natl Acad Sci USA 102:14266-71.

[6] Zhang XP, Liu F, Cheng Z, Wang W (2009) Cell fate decision mediated by p53 pulses. Proc Natl Acad Sci USA 106:12245-50.

[7] Neumaier T, Swenson J, Pham C, Polyzos A, Lo AT, et al. (2012) Evidence for formation of DNA repair centers and dose-response nonlinearity in human cells. Proc Natl Acad Sci USA 109:443-8.

[8] Stewart RD (2001) Two-lesion kinetic model of double-strand break rejoining and cell killing. Radiat Res 156:365-78.
