# Supplementary material for: A Two-Step Mechanism for Cell Fate Decision by Coordination of Nuclear and Mitochondrial p53 Activities
Source: PLoS One. 2012 Jun 5;7(6):e38164. doi: 10.1371/journal.pone.0038164 (PMC3367989; doi:10.1371/journal.pone.0038164)
Supplement: Method S2 — Ordinary differential equations for the model. (PDF) [file pone.0038164.s007.pdf]

Method S2: Ordinary differential equations for the model

$$\frac{d[\mathrm{p53_n}]}{dt} = k_{\mathrm{p53cn}}[\mathrm{p53_c}] - k_{\mathrm{p53nc}}[\mathrm{p53_n}] - k_{\mathrm{p53u}}[\mathrm{Mdm2_n}][\mathrm{p53_n}] + k_{\mathrm{p53du}}[\mathrm{p53U_n}] - k_{\mathrm{dp53}}[\mathrm{p53_n}] \quad (1)$$

$$\frac{d[\mathrm{p53U_n}]}{dt} = k_{\mathrm{p53u}}[\mathrm{Mdm2_n}][\mathrm{p53_n}] - k_{\mathrm{p53uu}}[\mathrm{Mdm2_n}][\mathrm{p53U_n}] + k_{\mathrm{p53duu}}[\mathrm{p53UU_n}] - k_{\mathrm{p53du}}[\mathrm{p53U_n}] - k_{\mathrm{p53unc}}[\mathrm{p53U_n}] - k_{\mathrm{dp53u}}[\mathrm{p53U_n}] \quad (2)$$

$$\frac{d[\mathrm{p53UU_n}]}{dt} = k_{\mathrm{p53uu}}[\mathrm{Mdm2_n}][\mathrm{p53U_n}] - k_{\mathrm{p53duu}}[\mathrm{p53UU_n}] - k_{\mathrm{dp53uu}}[\mathrm{p53UU_n}] \quad (3)$$

$$\frac{d[\mathrm{Mdm2_n}]}{dt} = k_{\mathrm{Mdm2Pcn}}[\mathrm{Mdm2P_c}] - k_{\mathrm{Mdm2nc}}[\mathrm{Mdm2_n}] - k_{\mathrm{dMdm2n}}[\mathrm{Mdm2_n}] \quad (4)$$

$$\frac{d[\mathrm{mdm2\ mRNA}]}{dt} = k_{\mathrm{smdm2}} + k_{\mathrm{mdm2}}\frac{[\mathrm{p53_n}]^4}{[\mathrm{p53_n}]^4 + J_{\mathrm{mdm2}}^4} - k_{\mathrm{dmdm2}}[\mathrm{mdm2\ mRNA}] \quad (5)$$

$$\frac{d[\mathrm{Mdm2_c}]}{dt} = k_{\mathrm{Mdm2}}[\mathrm{mdm2\ mRNA}] - k_{\mathrm{mdm2cp}}[\mathrm{Mdm2_c}]\frac{1}{1 + [\mathrm{p53_n}]} + k_{\mathrm{mdm2cdp}}[\mathrm{Mdm2P_c}] - k_{\mathrm{dMdm2c}}[\mathrm{Mdm2_c}] \quad (6)$$

$$\frac{d[\mathrm{Mdm2P_c}]}{dt} = k_{\mathrm{mdm2cp}}[\mathrm{Mdm2_c}]\frac{1}{1 + [\mathrm{p53_n}]} - k_{\mathrm{mdm2cdp}}[\mathrm{Mdm2P_c}] - k_{\mathrm{Mdm2Pcn}}[\mathrm{Mdm2P_c}] + k_{\mathrm{Mdm2nc}}[\mathrm{Mdm2_n}] - k_{\mathrm{dMdm2c}}[\mathrm{Mdm2P_c}] \quad (7)$$

$$\frac{d[\mathrm{p53_c}]}{dt} = k_{\mathrm{sp53}} - k_{\mathrm{p53cn}}[\mathrm{p53_c}] + k_{\mathrm{p53nc}}[\mathrm{p53_n}] - k_{\mathrm{p53u}}[\mathrm{Mdm2_{ct}}][\mathrm{p53_c}] + k_{\mathrm{p53du}}[\mathrm{p53U_c}] - k_{\mathrm{dp53}}[\mathrm{p53_c}] \quad (8)$$

$$\frac{d[\mathrm{p53U_c}]}{dt} = k_{\mathrm{p53u}}[\mathrm{Mdm2_{ct}}][\mathrm{p53_c}] - k_{\mathrm{p53uu}}[\mathrm{Mdm2_{ct}}][\mathrm{p53U_c}] + k_{\mathrm{p53duu}}[\mathrm{p53UU_c}] - k_{\mathrm{p53du}}[\mathrm{p53U_c}] + k_{\mathrm{p53unc}}[\mathrm{p53U_n}] - k_{\mathrm{dp53u}}[\mathrm{p53U_c}] - k_{\mathrm{sp53m}}[\mathrm{p53U_c}] - k_{\mathrm{p53m}}[\mathrm{p53U_c}]\frac{N_{\mathrm{DSB}}}{N_{\mathrm{DSB}} + J_{\mathrm{p53m}}} \quad (9)$$

$$\frac{d[\mathrm{p53UU_c}]}{dt} = k_{\mathrm{p53uu}}[\mathrm{Mdm2_{ct}}][\mathrm{p53U_c}] - k_{\mathrm{p53duu}}[\mathrm{p53UU_c}] - k_{\mathrm{dp53uu}}[\mathrm{p53UU_c}] \quad (10)$$

$$\frac{d[\mathrm{DYRK2_n}]}{dt} = k_{\mathrm{DYRKcn}}\frac{N_{\mathrm{DSB}}}{N_{\mathrm{DSB}} + J_{\mathrm{DYRK2}}}(\mathrm{DYRK2_t} - [\mathrm{DYRK2_n}]) - k_{\mathrm{DYRKnc}}[\mathrm{DYRK2_n}] \quad (11)$$

$$\frac{d[\mathrm{p53\ killer}]}{dt} = k_{\mathrm{pp53}}[\mathrm{DYRK2_n}]\frac{[\mathrm{p53\ arrester}]}{[\mathrm{p53\ arrester}] + J_{\mathrm{pp53}}} - k_{\mathrm{dpp53}}\frac{[\mathrm{p53\ killer}]}{[\mathrm{p53\ killer}] + J_{\mathrm{dpp53}}} \quad (12)$$

$$\frac{d[\mathrm{p53_m}]}{dt} = k_{\mathrm{sp53m}}[\mathrm{p53U_c}] + k_{\mathrm{p53m}}[\mathrm{p53U_c}]\frac{N_{\mathrm{DSB}}}{N_{\mathrm{DSB}} + J_{\mathrm{p53m}}} - V_{\mathrm{bak1}}[\mathrm{p53_m}]\frac{[\mathrm{Bak}]}{[\mathrm{Bak}] + J_{\mathrm{bak1}}} - k_{\mathrm{dp53m}}[\mathrm{p53_m}] \quad (13)$$

$$\frac{d[\text{p21 mRNA}]}{dt} = k_{\text{sp21}} + k_{\text{p21}}\frac{[\text{p53 arrester}]^4}{[\text{p53 arrester}]^4 + J_{\text{p21}}^4} - k_{\text{dp21}}[\text{p21 mRNA}] \quad (14)$$

$$\frac{d[\text{p21}]}{dt} = k_{\text{P21}}[\text{p21 mRNA}] - k_{\text{dP21s}}[\text{p21}] - k_{\text{dP21}}[\text{Casp3}]\frac{[\text{p21}]}{[\text{p21}] + J_{\text{dP21}}} \quad (15)$$

$$\frac{d[\text{puma mRNA}]}{dt} = k_{\text{spuma}} + k_{\text{puma}}\frac{[\text{p53 killer}]^4}{[\text{p53 killer}]^4 + J_{\text{puma}}^4} - k_{\text{dpuma}}[\text{puma mRNA}] \quad (16)$$

$$\frac{d[\text{PUMA}]}{dt} = k_{\text{Puma}}[\text{puma mRNA}] - k_{\text{dPuma}}[\text{PUMA}] - k_{\text{bak2}}[\text{PUMA}]\frac{[\text{Bak}]}{[\text{Bak}] + J_{\text{bak2}}} \quad (17)$$

$$\frac{d[\text{Bak}^*]}{dt} = k_{\text{bak1}}[\text{p53}_{\text{m}}]\frac{[\text{Bak}]}{[\text{Bak}] + J_{\text{bak1}}} + k_{\text{bak2}}[\text{PUMA}]\frac{[\text{Bak}]}{[\text{Bak}] + J_{\text{bak2}}} + k_{\text{bak3}}[\text{Casp3}]\frac{[\text{Bak}]}{[\text{Bak}] + J_{\text{bak3}}} - k_{\text{dbak}}[\text{Bak}^*] \quad (18)$$

$$\frac{d[\text{Apaf1}]}{dt} = k_{\text{sApaf1}} + k_{\text{Apaf1}}\frac{[\text{E2F1}]^3}{[\text{E2F1}]^3 + J_{\text{Apaf1}}^3} - k_{\text{dApaf1}}[\text{Apaf1}] - n(k_{\text{aApop}}[\text{CytoC}]^n[\text{Apaf1}]^n - k_{\text{deApop}}[\text{Apop}]) \quad (19)$$

$$\frac{d[\text{CytoC}]}{dt} = k_{\text{CytoC}}[\text{Bak}](\text{CytoC}_{\text{t}} - [\text{CytoC}]) - k_{\text{dCytoC}}[\text{CytoC}] - n(k_{\text{aApop}}[\text{CytoC}]^n[\text{Apaf1}]^n - k_{\text{deApop}}[\text{Apop}]) \quad (20)$$

$$\frac{d[\text{Apop}]}{dt} = k_{\text{aApop}}[\text{CytoC}]^n[\text{Apaf1}]^n - k_{\text{deApop}}[\text{Apop}] - k_{\text{dApop}}[\text{Apop}] \quad (21)$$

$$\frac{d[\text{Casp3}]}{dt} = k_{\text{Casp3}}[\text{Apop}](\text{Casp3}_{\text{t}} - [\text{Casp3}]) - k_{\text{dCaps3}}[\text{Casp3}] \quad (22)$$

$$[\text{p53}_{\text{n}}] = [\text{p53 arrester}] + [\text{p53 killer}]$$

$$[\text{Mdm2}_{\text{ct}}] = [\text{Mdm2}_{\text{c}}] + [\text{Mdm2P}_{\text{c}}]$$

$$[\text{Bak}] = \text{Bak}_{\text{t}} - [\text{Bak}^*]$$

$$[\text{E2F1}] = G(0.5, [\text{p21}], 0.1, 0.1)$$

$$k_{\text{dMdm2n}} = k_{\text{dMdm2c}} + k_{\text{dMdm2}}\frac{N_{\text{DSB}}}{N_{\text{DSB}} + J_{\text{dm}}}$$

$$G(u,v,x,y) = \frac{2uy}{v - u + vx + uy + \sqrt{(v-u+vx+uy)^2 - 4(v-u)uy}}$$
