# Supplementary material for: A Two-Step Mechanism for Cell Fate Decision by Coordination of Nuclear and Mitochondrial p53 Activities
Source: PLoS One. 2012 Jun 5;7(6):e38164. doi: 10.1371/journal.pone.0038164 (PMC3367989; doi:10.1371/journal.pone.0038164)
Supplement: Table S1 — Description and initial values of the model variables. (PDF) [file pone.0038164.s008.pdf]

Table S1: Description and Initial values of the model variables

Variable	Description	Initial values
N_{DSB}	Number of total double-strand breaks	0
$N_{\mathrm{D_1}}$	Number of simple double-strand breaks	0
$N_{\mathrm{C_1}}$	Number of simple DSBs in complex with repair proteins	0
$N_{\mathrm{F_1}}$	Number of fixed simple double-strand breaks	0
$N_{\mathrm{D_2}}$	Number of complex double-strand breaks	0
$N_{\mathrm{C_2}}$	Number of complex DSBs in complex with repair proteins	0
$N_{\mathrm{F_2}}$	Number of fixed complex double-strand breaks	0
$[\mathrm{p53_n}]$	Concentration of nuclear un-ubiquitinated p53	0.13
$[\mathrm{p53U_n}]$	Concentration of nuclear mono-ubiquitinated p53	0.05
$[\mathrm{p53UU_n}]$	Concentration of nuclear poly-ubiquitinated p53	0.004
$[\mathrm{Mdm2_n}]$	Concentration of nuclear Mdm2	0.83
[mdm2 mRNA]	Concentration of mdm2 mRNA	0.09
$[\mathrm{Mdm2_c}]$	Concentration of cytoplasmic Mdm2	0.04
$[\mathrm{Mdm2P_c}]$	Concentration of cytoplasmic phosphorylated Mdm2	0.03
$[\mathrm{p53_c}]$	Concentration of cytoplasmic un-ubiquitinated p53	0.06
$[\mathrm{p53U_c}]$	Concentration of cytoplasmic mono-ubiquitinated p53	0.31
$[\mathrm{p53UU_c}]$	Concentration of cytoplasmic poly-ubiquitinated p53	0.002
$[\mathrm{DYRK2_n}]$	Concentration of nuclear DYRK2	0
[p53 arrester]	Concentration of primarily phosphorylated p53	0.13
[p53 killer]	Concentration of further phosphorylated p53 at Ser46	0
$[\mathrm{p53_m}]$	Concentration of mitochondrial un-ubiquitinated p53	0.001
[p21 mRNA]	Concentration of p21 mRNA	0.016
[p21]	Concentration of p21	0.1
[puma mRNA]	Concentration of puma mRNA	0.02
[PUMA]	Concentration of puma	0.01
[Bak*]	Concentration of activated Bak	0.016
[Apaf1]	Concentration of Apaf1	1.95
[CytoC]	Concentration of cytoplasmic cytochrome c	0.025
[Apop]	Concentration of Apoptosome	0
[Casp3]	Concentration of active caspase 3	0
