# Supplementary material for: A Two-Step Mechanism for Cell Fate Decision by Coordination of Nuclear and Mitochondrial p53 Activities
Source: PLoS One. 2012 Jun 5;7(6):e38164. doi: 10.1371/journal.pone.0038164 (PMC3367989; doi:10.1371/journal.pone.0038164)
Supplement: Table S2 — Description and values of the model parameters. (PDF) [file pone.0038164.s009.pdf]

Table S2: Description and values of the model parameters

Rate Constant	Description	Value	Reference
$T_{1/2}$	Time for half of total DSBs to be generated	5	[1]
N_{RPt}	Number of repair proteins	20	[2, 3]
k_{cross}	DSB binary mismatch rate	0.001	[2, 3]
k_{fb1}	Association rate of repair proteins in fast kinetics	2	[2, 3]
k_{rb1}	Dissociation rate of repair proteins in fast kinetics	0.5	[2, 3]
k_{fix1}	DSB ligation rate in fast kinetics	0.03	[2, 3]
k_{fb2}	Association rate of repair proteins in slow kinetics	0.2	[2, 3]
k_{rb2}	Dissociation rate of repair proteins in slow kinetics	0.05	[2, 3]
k_{fix2}	DSB ligation rate in slow kinetics	0.003	[2, 3]
k_{p53cn}	Nuclear import rate of cytoplasmic p53	14	Estimated
k_{p53nc}	Nuclear export rate of nuclear p53	0.5	Estimated
k_{p53u}	Ubiquitination rate of p53	8.8	[4]
k_{p53du}	Deubiquitination rate of p53U	2.5	[4]
k_{dp53}	Degradation rate of p53	0.0055	[4]
k_{p53uu}	Ubiquitination rate of p53U	1	[4]
k_{p53duu}	Deubiquitination rate of p53UU	2.5	[4]
k_{p53unc}	Nuclear export rate of nuclear p53U	14	Estimated
k_{dp53u}	Degradation rate of p53U	0.0055	[4]
k_{dp53uu}	Degradation rate of p53UU	8	[4]
k_{Mdm2Pcn}	Nuclear import rate of cytoplasmic phosphorylated Mdm2	14	[4]
k_{Mdm2nc}	Nuclear export rate of nuclear Mdm2	0.5	[4]
k_{dMdm2}	DSBs-dependent degradation rate of nuclear Mdm2	0.05	[4]
k_{smdm2}	Basal transcription rate of Mdm2	0.0009	[2]
k_{mdm2}	p53-dependent transcription rate of Mdm2	0.0375	[2]
J_{mdm2}	Michaelis constant of p53-dependent mdm2 transcription	3	[2]
k_{dmdm2}	Degradation rate of mdm2 mRNA	0.01	[2]
k_{Mdm2}	Translation rate of mdm2 mRNA	0.1	[2]
k_{dMdm2c}	Degradation rate of cytoplasmic Mdm2	0.01	[4]
k_{Mdm2cp}	Phosphorylation rate of cytoplasmic Mdm2	1	Estimated
k_{Mdm2cdp}	Dephosphorylation rate of cytoplasmic phosphorylated Mdm2	1	Estimated
k_{sp53}	Production rate of p53	0.055	[4]
k_{sp53m}	Basal mitochondrial translocation rate of p53	0.0001	Estimated
k_{p53m}	DSBs-dependent mitochondrial translocation rate of p53	0.03	Estimated
J_{p53m}	Michaelis constant of DSBs-dependent p53 mitochondrial translocation	212.5	Estimated

Table S2: Description and values of the model parameters-Continued

Rate Constant	Description	Value	Reference
k_{DYRKcn}	Nuclear import rate of cytoplasmic DYRK2	0.00015	[5]
J_{DYRK2}	Michaelis constant of DSBs-dependent DYRK2 nuclear import	89	Estimated
$\mathrm{DYRK2_t}$	Total concentration of DYRK2	8	[5]
k_{DYRKnc}	Nuclear export rate of nuclear DYRK2	0.0003	[5]
k_{pp53}	Rate constant of p53 arrester phosphorylation	0.03	[5]
J_{pp53}	Michaelis constant of p53 arrester phosphorylation	0.1	[5]
k_{dpp53}	Rate constant of p53 killer dephosphorylation	0.005	[5]
J_{dpp53}	Michaelis constant of p53 killer dephosphorylation	0.5	[5]
J_{dm}	Michaelis constant of DSBs-dependent nuclear Mdm2 degradation	59.5	Estimated
k_{dp53m}	Degradation rate of mitochondrial p53	0.01	Estimated
k_{bak1}	Mitochondrial p53-dependent activation rate of Bak	0.015	Estimated
J_{bak1}	Michaelis constant of mitochondrial p53-dependent Bak activation	0.3	Estimated
k_{sp21}	Basal transcription rate of p21	0.0008	Estimated
k_{p21}	p53-dependent transcription rate of p21	0.026	Estimated
J_{p21}	Michaelis constant of p53-dependent p21 transcription	0.3	Estimated
k_{dp21}	Degradation rate of p21 mRNA	0.1	Estimated
k_{P21}	Translation rate of p21 mRNA	0.03	Estimated
k_{dP21s}	Basal degradation rate of p21	0.005	Estimated
k_{dP21}	Casp3-dependent degradation rate of p21	0.2	Estimated
J_{dP21}	Michaelis constant of Casp3-dependent p21 degradation	0.3	Estimated
k_{spuma}	Basal transcription rate of puma	0.001	Estimated
k_{puma}	p53-dependent transcription rate of puma	0.02	Estimated
J_{puma}	Michaelis constant of p53-dependent puma transcription	0.2	Estimated
k_{dpuma}	Degradation rate of puma mRNA	0.05	Estimated
k_{Puma}	Translation rate of puma mRNA	0.01	Estimated
k_{dPuma}	Basal degradation rate of PUMA	0.005	Estimated
k_{bak2}	PUMA-dependent activation rate of Bak	0.015	Estimated
J_{bak2}	Michaelis constant of PUMA-dependent Bak activation	0.3	Estimated
k_{bak3}	Casp3-dependent activation rate of Bak	0.3	Estimated
J_{bak3}	Michaelis constant of Casp3-dependent Bak activation	0.3	Estimated
$\mathrm{Bak_t}$	Total concentration of Bak	3	Estimated
k_{dbak}	Deactivation rate of Bak	0.01	Estimated

Table S2: Description and values of the model parameters-Continued

Rate Constant	Description	Value	Reference
k_{sApaf1}	Basal production rate of Apaf1	0.01	[6]
k_{Apaf1}	E2F1-dependent production rate of Apaf1	0.09	[6]
J_{Apaf1}	Michaelis constant of E2F1-dependent Apaf1 production	0.3	[6]
k_{dApaf1}	Degradation rate of Apaf1	0.05	[6]
k_{CytoC}	Bak-dependent release rate of mitochondrial cytochrome c	0.03	Estimated
k_{dCytoC}	Mitochondrial influx rate of cytochrome c	0.1	Estimated
$\mathrm{CytoC_t}$	Total concentration of cytochrome c	5	[7]
k_{aApop}	Activation rate of apoptosome	0.2	Estimated
k_{deApop}	Inactivation rate of apoptosome	8	Estimated
k_{dApop}	Degradation rate of apoptosome	0.002	Estimated
k_{Casp3}	Activation rate of caspase 3	2	[8]
k_{dCasp3}	Inactivation rate of caspase 3	0.02	[8]
$\mathrm{Casp3_t}$	Total concentration of caspase 3	3	[8]

---

[1] Neumaier T, Swenson J, Pham C, Polyzos A, Lo AT, et al. (2012) Evidence for formation of DNA repair centers and dose-response nonlinearity in human cells. Proc Natl Acad Sci USA 109: 443-448.

[2] Ma L, Wagner J, Rice JJ, Hu W, Levine AJ, et al. (2005) A plausible model for the digital response of p53 to DNA damage. Proc Natl Acad Sci USA 102:14266-14271.

[3] Zhang XP, Liu F, Cheng Z, W. Wang (2009) Cell fate decision mediated by p53 pulses. Proc Natl Acad Sci USA 106:12245-12250.

[4] Ciliberto A, Novak B, Tyson JJ (2005) Steady states and oscillations in the p53/Mdm2 network. Cell Cycle 4:488-493.

[5] Zhang T, Brazhnik P, Tyson JJ (2009) Computational analysis of dynamical responses to the intrinsic pathway of programmed cell death. Biophys J 97:415-434.

[6] Zhang T, Brazhnik P, Tyson JJ (2007) Exploring mechanisms of the DNA-damage response: p53 pulses and their possible relevance to apoptosis. Cell Cycle 6:85-94.

[7] Zhang XP, Liu F, Wang W (2010) Coordination between Cell Cycle Progression and Cell Fate Decision by the p53 and E2F1 Pathways in Response to DNA Damage. J Biol Chem 285:31571-31580.

[8] Zhang X P, Liu F, Wang W (2011) Two-phase dynamics of p53 in the DNA damage response. Proc Natl Acad Sci USA 108:8990-8995.
